# Unravelling the Distinct Effects of Systolic and Diastolic Blood Pressure Using Mendelian Randomisation

**DOI:** 10.3390/genes13071226

**Published:** 2022-07-09

**Authors:** Nhu Ngoc Le, Tran Q. B. Tran, Stefanie Lip, Linsay McCallum, John McClure, Anna F. Dominiczak, Dipender Gill, Sandosh Padmanabhan

**Affiliations:** 1 Institute of Cardiovascular and Medical Sciences, University of Glasgow, Glasgow G12 8TA, UK; 2494395l@student.gla.ac.uk (N.N.L.); 2119810t@student.gla.ac.uk (T.Q.B.T.); stefanie.lip@glasgow.ac.uk (S.L.); linsay.mccallum@glasgow.ac.uk (L.M.); john.d.mcclure@glasgow.ac.uk (J.M.); anna.dominiczak@glasgow.ac.uk (A.F.D.); 2 Department of Epidemiology and Biostatistics, School of Public Health, Imperial College London, London W2 1PG, UK; dipender.gill@imperial.ac.uk

**Keywords:** blood pressure, systolic, diastolic, Mendelian randomization

## Abstract

A true discrepancy between the effect of systolic blood pressure (SBP) and diastolic blood pressure (DBP) on cardiovascular (CV) outcomes remains unclear. This study performed two-sample Mendelian randomization (MR) using genetic instruments that exclusively predict SBP, DBP or both to dissect the independent effect of SBP and DBP on a range of CV outcomes. Genetic predisposition to higher SBP and DBP was associated with increased risk of coronary artery disease (CAD), myocardial infarction (MI), stroke, heart failure (HF), atrial fibrillation (AF), chronic kidney disease (CKD) and type 2 diabetes mellitus (T2DM). Genetically proxied SBP exclusively was associated with CAD (OR 1.18, 95% CI: 1.03–1.36, per 10 mmHg), stroke (1.44[1.28–1.62]), ischemic stroke (1.49[1.30–1.69]), HF (1.41[1.20–1.65]), AF (1.28[1.15–1.43]), and T2DM (1.2[1.13–1.46]). Genetically proxied DBP exclusively was associated with stroke (1.21[1.06–1.37], per 5 mmHg), ischemic stroke (1.24[1.09–1.41]), stroke small-vessel (1.35[1.10–1.65]) and CAD (1.19[1.00–1.41]). Multivariable MR using exclusive SBP and DBP instruments showed the predominant effect of SBP on CAD (1.23[1.05–1.44], per 10 mmHg), stroke (1.39[1.20–1.60]), ischemic stroke (1.44[1.25–1.67]), HF (1.42[1.18–1.71]), AF (1.26[1.10–1.43]) and T2DM (1.31[1.14–1.52]). The discrepancy between effects of SBP and DBP on outcomes warrants further studies on underpinning mechanisms which may be amenable to therapeutic targeting.

## 1. Introduction

High blood pressure (BP) is recognized as the most common modifiable risk factor for cardiovascular diseases (CVDs) and related disability. More than 1 billion people worldwide experience high blood pressure [1], which accounts for more than 20% of CVD [2]. A 10 mmHg decrease in systolic blood pressure (SBP) was found to be associated with a 5% to 20% reduction in risk of coronary artery disease (CAD) and a 25% to 30% reduction in the risk of stroke [3], indicating BP lowering is one of the most effective strategies to reduce the burden of CVD. Blood pressure is represented by systolic and diastolic components, both of which are considered for a diagnosis of hypertension. Whilst both show direct associations with incident cardiovascular disease [4,5], SBP alone is included in CVD risk prediction tools. This reflects the current parsimonious notion that SBP alone is sufficient to capture hypertension-related CVD risk, especially given the global ageing population demographics [6]. Traditionally, systolic blood pressure (SBP) and diastolic blood pressure (DBP) represent cardiac output and peripheral vascular resistance, respectively. SBP rises with age linearly, while DBP increases until around age 50 years and falls thereafter accompanied by wider pulse pressure. The rise in BP with age transitions from a primarily vasoconstrictive mechanism in resistance arteries and arterioles affects both SBP and DBP in the young to large artery stiffening and loss of vascular compliance in older individuals that increases SBP but decreases DBP. Epidemiologically, DBP is more closely associated with coronary heart disease (CHD) development in the young, whereas in those over 60 years SBP is more predictive [7]. A DBP J-curve relationship has been observed where low DBP is associated with increased CV risk in the presence of coronary artery disease and attributed to limited coronary flow reserve [5,8,9], though not consistently [4], and refuted in recent Mendelian randomization studies [10,11]. Compared with DBP, raised SBP had a greater effect on angina, myocardial infarction and peripheral arterial disease, whereas raised DBP had a greater effect on abdominal aortic aneurysm [4]. There is considerable epidemiological evidence associating hypertension with heart failure but evidence for a causal role is less clear; although fibrosis, ventricular noncompliance, hypertrophy and ischemia are common features of HF which can all be impacted by hypertension. Genome-wide association studies (GWAS) have uncovered >1500 single nucleotide polymorphisms associated with SBP and DBP (with a minority of SNPs exclusively associated with either SBP or DBP) and have transformed our understanding of blood pressure genetics. This has paved the way for Mendelian randomisation (MR) studies which leverage the independent assortment of genotypes to minimise confounding and reverse causation [12] to show a causal relationship between SBP and coronary artery disease [13,14], stroke (ischemic stroke and deep intracerebral hemorrhage) [14,15] and valvular heart disease [16]. Applying non-linear MR, two studies ruled out a J-shaped relationship between DBP and CAD [10,14] and stroke [14]. It is unclear if a true dichotomy between SBP and DBP exists where separate pathophysiological pathways affect outcomes independently. However, if such a dichotomy exists, then understanding its basis may lead to precision hypertension management. To address this question, we performed MR studies using genetic instruments that exclusively predict SBP, DBP or both to dissect the independent effect of SBP and DBP on a range of cardiovascular outcomes.

## 2. Methods

### 2.1. Two-Sample MR Analysis

We performed two-sample MR using GWAS summary data. Data used in this study are available from public repositories and are summarised in Table 1. Genetic associations of single-nucleotide polymorphisms (SNPs) with SBP and DBP were obtained from a GWAS meta-analysis for BP traits [17]. Genetic associations with outcomes were obtained from publicly available genetic consortia. The β-coefficients obtained from these published GWAS studies had been corrected for population stratification by inclusion of principal components into their analysis [17,18,19,20,21,22,23,24]. This MR analysis was reported as per the STROBE-MR guidelines [25] (Supplementary checklist in the Data Supplement).

### 2.2. Genetic Instruments for Blood Pressure

**Figure 1:**
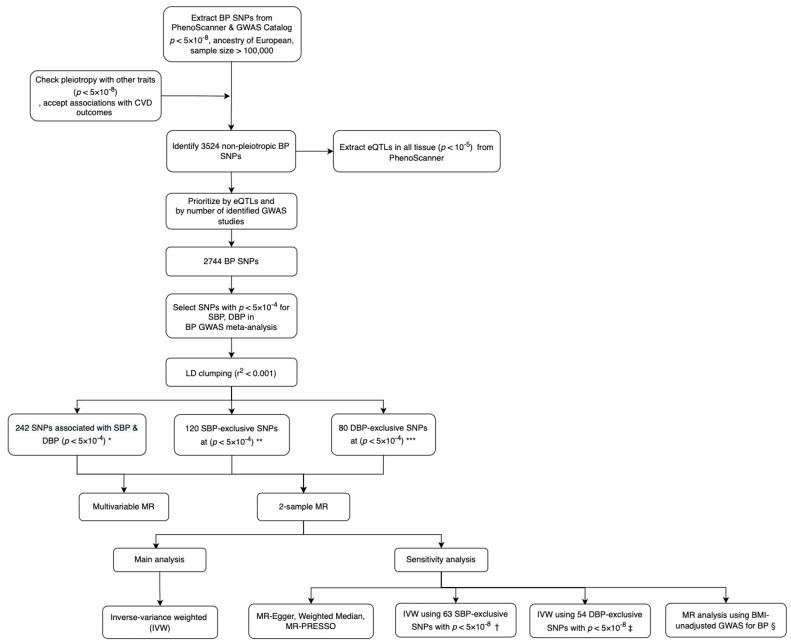
Flowchart illustrating the selection criteria for genetic instruments in this study. BP indicates blood pressure; SBP, systolic blood pressure; DBP, diastolic blood pressure; SNP, single nucleotide polymorphism; GWAS, Genome-wide association study; CVD, cardiovascular disease; eQTL, expression quantitative trait loci; LD, linkage disequilibrium; MR, Mendelian randomization; BMI, body mass index. * 242 SNPs associated with both SBP and DBP (*p* < 5 × 10^−4^), identified in a BP GWAS meta-analysis study [17]. ** 120 SNPs associated with SBP but not associated with DBP (*p* < 5 × 10^−4^). *** 80 SNPs associated with DBP but not associated with SBP (*p* < 5 × 10^−4^). † 63 SNPs associated with SBP (*p* < 5 × 10^−8^) but not associated with DBP (*p* > 5 × 10^−4^). ‡ 54 SNPs associated with DBP (*p* < 5 × 10^−8^) but not with SBP (*p* > 5 × 10^−4^). § MR analysis unadjusted for body mass index, using summary statistics from UK BioBank data.

All the data for instrumental variable selection were obtained from the GWAS catalogue [26] and PhenoScanner [27]. Genetic instruments were selected for the MR analysis based on two-stage inclusion criteria. In stage 1, we selected SNPs that were associated with any BP trait (SBP, DBP, pulse pressure (PP), hypertension (HTN)) at *p*-value < 5 × 10^−8^ in GWAS studies on European participants with a sample size > 100,000 through a review of the GWAS catalog and PhenoScanner. We excluded SNPs pleiotropic for other phenotypes except cardiovascular outcomes (defined by associations at *p*-value < 5 × 10^−8^) through review of all GWAS studies available in the GWAS catalog and PhenoScanner [26,27]. Then we prioritized SNPs for inclusion which satisfied at least one of the following criteria: (i) SNPs were associated with cis- or trans-gene expression in one or multiple tissues at *p*-value < 5 × 10^−5^ (i.e., expression quantitative trait loci, eQTL); (ii) SNPs were associated with BP traits in more than one GWAS. In stage 2, we grouped the SNPs into three mutually exclusive sets using GWAS results from Evangelou et al. [17], the largest GWAS of blood pressure traits with publicly accessible summary data, where we focused on SBP and DBP association results and removed SNPs that had a *p*-value > 5 × 10^−4^ in this study. This resulted in a set of SNPs that attained a *p*-value < 5 × 10^−8^ for any BP trait in a large GWAS study and had an SBP or DBP *p*-value < 5 × 10^−4^ in Evangelou et al. and were grouped in SBP-exclusive, DBP-exclusive and SBP +DBP groups. Each SBP-exclusive SNP had *p*-value < 5 × 10^−4^ for SBP and *p*-value > 5 × 10^−4^ for DBP in Evangelou et al. and a similar principle applied to DBP-exclusive SNPs. We further used a more stringent *p*-value < 5 × 10^−8^ instead of 5 × 10^−4^ to select SNPs for sensitivity analysis. The selection criteria for genetic instruments in this MR study are illustrated in Figure 1. Palindromic SNPs with effect allele frequency close to 0.5 (>0.42 and <0.58) were removed from the analysis and to ensure that genetic instruments were independent, we performed clumping at r^2^ < 0.001. This resulted in 242 independent SNPs associated with both SBP and DBP (Set 1); 120 SBP-exclusive SNPs (Set 2); and 80 DBP-exclusive SNPs (Set 3). For sensitivity analysis, we obtained 63 SBP-exclusive SNPs (SBP *p* < 5 × 10^−8^, DBP *p* > 5 × 10^−4^) (Set 2′), and 54 DBP-exclusive SNPs (DBP *p* < 5 × 10^−8^, SBP *p* > 5 × 10^−4^) (Set 3′) (Tables S1–S6).

The summary statistics for selected SNPs were obtained from a GWAS meta-analysis for BP traits, Evangelou et al. [17], on 757,601 individuals of European ancestry. This study comprises a fixed-effects inverse variance weighted meta-analysis of genotyped and imputed SNPs from the International Consortium for Blood Pressure (ICBP) and the UK Biobank. The ICBP GWAS data consists of 77 cohorts for 299,024 individuals of European ancestry genotyped with various arrays and imputed to either the 1000 Genomes Reference Panel or the Haplotype Reference Consortium (HRC) panel. UK Biobank is a cohort study of 502,519 people aged 40–69 years who are mainly of British ancestry. The participants were genotyped by a customized array and imputed to the HRC panel. Both ICBP and UKBB GWAS were adjusted for age, age^2^, sex and body mass index (BMI), and included study-level genomic control to account for population structure. These studies also corrected for observed BP based on hypertension medication status. The pooled mean (standard deviation) of SBP and DBP were 138.4 (20.1) and 82.8 (11.2) mmHg, respectively [17].

### 2.3. Outcomes

Outcomes selected were a range of CVD outcomes related to hypertension, including coronary artery disease (CAD), myocardial infarction (MI), stroke, heart failure (HF), atrial fibrillation (AF), chronic kidney disease (CKD) and type 2 diabetes mellitus (T2DM) [4,28,29,30,31,32,33,34]. Genetic associations with these outcomes were obtained from the most appropriate disease GWAS from publicly available genetic consortia, which consist of the most similar populations with the GWAS for the exposure while minimizing sample overlap. Details of the GWAS consortium are listed in Table 1. 

### 2.4. Ethical Approval

This study only used publicly available data. Ethical approval for each of the studies can be found in the original publications.

### 2.5. Statistical Analysis

MR analysis relies on three assumptions, including that the genetic instruments were robustly associated with the exposure of interest, were independent of potential confounders and were associated with the outcomes only via their association with the exposure. In this MR study, we assessed the strength of genetic instruments by calculating the F statistics [35]. We used the GWAS catalogue [26] and PhenoScanner [27], which provide a curated database of publicly available large-scale GWAS, to investigate pleiotropic associations of BP SNPs. 

To estimate the causal effects of BP traits on the odds of the outcomes we performed two-sample MR analysis using inverse variance weighted (IVW) with multiplicate random effects. Three sets of genetic instruments—SBP-and-DBP-associated SNPs (set 1), SBP-exclusive SNPs (set 2) and DBP-exclusive SNPs (set 3)—were used for the two-sample MR analysis to differentiate the causal effects of SBP and DBP on CV outcomes. The proportion of variance (R^2^) in the exposure trait explained by each genetic variant was calculated [36]. Estimates of the effects of each BP trait on outcomes are odds ratios per 10 mmHg increase in genetically predicted SBP and are odds ratios per 5 mmHg increase in genetically predicted DBP. With consideration of multiple testing and the similar aetiology of several outcomes included in this study, a Bonferroni-corrected *p*-value of 0.004 (0.05/(2 BP traits × 6 outcomes)) was considered strong evidence, whereas *p*-value < 0.05 but > 0.004 was considered suggestive evidence. To assess the heterogeneity of the effects we used the Cochran Q test [37] and scatterplots of the SNP effects on the exposure against SNP effects on the outcome. To detect horizontal pleiotropy effects of the instruments, we evaluated the Egger intercept in MR Egger regression; a significant deviation of the intercept from zero indicates possible horizontal pleiotropy [38]. The MR Egger method relaxes the MR assumptions, allowing for directional pleiotropy but requiring the Instrument Strength Independent of Direct Effect (InSIDE) assumption [38]. The InSIDE assumption is satisfied when the pleiotropic effects of genetic variants on the outcome are not correlated with their associations with the exposure [38]. A funnel plot of the MR estimate against its precision was conducted to detect directional pleiotropy. To identify if a single SNP is driving the association, we performed leave-one-out analysis by leaving each SNP out of the MR analysis in turn [39]. Moreover, to explore the independent effects of SBP and DBP on outcomes, we conducted multivariable Mendelian randomization (MVMR) analyses in SBP-exclusive + DBP-exclusive SNP set. We used the Sanderson–Windmeijer multivariate F-statistic to assess the conditional instrument strength and the modified Cochran’s Q-statistics to measure the heterogeneity [40]. A conditional F-statistic greater than 10 is conventionally used as a threshold for sufficient instrument strength. The IVW MVMR method was performed to estimate the independent causal effects of SBP and DBP on outcomes. Robust causal estimates were obtained through Q-statistic minimization, accounting for potential violation in the MVMR assumptions [40].

As the IVW method assumes that all variants satisfy the assumptions of MR analysis or that the average pleiotropic effect across genetic variants is zero [41], we conducted sensitivity analysis using weighted median [42], and Mendelian Randomization-Pleiotropy Residual Sum and Outlier (MR-PRESSO) [43] to assess the robustness of the results. These methods can provide reliable inferences when some genetic variants do not satisfy the assumptions. The MR-PRESSO method allows for the detection of variants with heterogeneous estimates which can be horizontal pleiotropic outliers. The detected variants were removed from the analysis and the IVW method was subsequently conducted without such variants [43]. The weighted median method, which takes a median of the variant-specific estimates, is robust to directional pleiotropy when at least half of the SNPs are valid instruments and was also performed [42]. Additionally, we performed the IVW MR method using subsets of SBP-exclusive and DBP-exclusive SNPs (set 2′ and 3′), in which we only selected SNPs that had significant associations with the traits at *p*-value < 5 × 10^−8^. As the GWAS study by Evangelou et al. [17] adjusted for the effect of the body-mass index (BMI) which could introduce collider bias, we conducted the MR analysis using genetic associations with BP in UK Biobank that did not include adjustment for BMI [18] available through MR-Base (MR-base id: ukb-a-360, ukb-a-359) [44]. 

MR analyses by IVW, weighted median and MR Egger were performed using R package TwoSampleMR [44]; MR-PRESSO was performed using R package MR-PRESSO; MVMR analyses were performed using R package MVMR [45]. All analyses were performed in R software version 4.1.2. 

## 3. Results

All the selected SNPs as genetic instruments had F statistics > 10, suggesting that bias due to weak instruments was unlikely to be influencing our conclusions. The total variance explained for the selected instruments is 2.82% and 2.54% for SBP and DBP, respectively.

**Figure 2:**
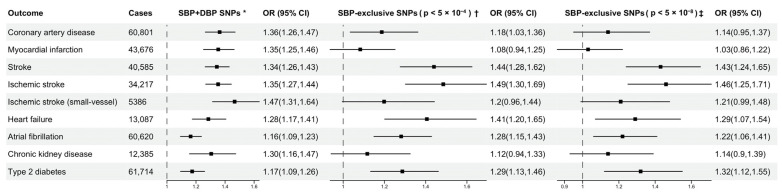
Mendelian randomization estimates (odds ratio with 95% CI per 10 mmHg increase in genetically predicted systolic blood pressure) from the inverse variance weighted method. SNP indicates single nucleotide polymorphism; OR, odd ratios; SBP, systolic blood pressure; DBP, diastolic blood pressure. * 242 SNPs associated with both SBP and DBP (*p* < 5 × 10^−4^), identified in a BP GWAS meta-analysis study [17]. † 120 SNPs associated with SBP (*p* < 5 × 10^−4^) but not associated with DBP. ‡ 63 SNPs associated with SBP (*p* < 5 × 10^−8^) but not associated with DBP (*p* > 5 × 10^−4^).

**Figure 3:**
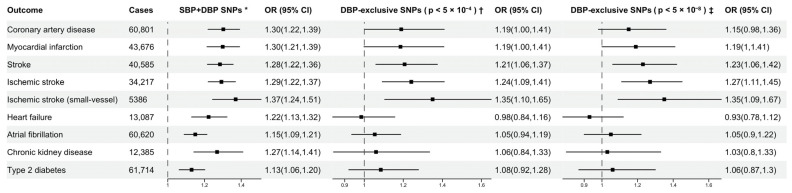
Mendelian randomization estimates (odds ratio with 95% CI per 5 mmHg increase in genetically predicted diastolic blood pressure) from the inverse variance weighted method. SNP indicates single nucleotide polymorphism; OR, odd ratios; SBP, systolic blood pressure; DBP, diastolic blood pressure. * 242 SNPs associated with both SBP and DBP (*p* < 5 × 10^−4^), identified in a BP GWAS meta-analysis study [17]. † 80 SNPs associated with DBP (*p* < 5 × 10^−4^) but not associated with SBP. ‡ 54 SNPs associated with DBP (*p* < 5 × 10^−8^) but not with SBP (*p* > 5 × 10^−4^).

Using SNPs associated with both SBP and DBP (set 1) as genetic instruments, we found that both SBP and DBP were positively associated with all CVD outcomes included in this study. In decreasing order of the IVW estimates, each genetically predicted 10 mmHg increase in SBP was associated with an odds ratio (OR) of 1.36 in CAD (95% CI, 1.26–1.47), 1.35 in MI (95% CI, 1.25–1.46), 1.34 in stroke (95% CI, 1.26–1.43), 1.30 in CKD (95% CI, 1.16–1.47), 1.28 in HF (95% CI, 1.17–1.41), 1.17 in T2DM (95% CI, 1.09–1.26) and 1.16 in AF (95% CI, 1.09–1.24) (Figure 2). A similar trend was observed in the associations of DBP with the outcomes, with the strongest associations being with CAD and MI, and the least strong associations being with AF and T2DM (Figure 3). The MR-Egger intercept test did not detect significant directional pleiotropy (Tables S7 and S8). Estimates from the weighted median and MR-PRESSO methods were consistent in direction and magnitude with the results from the main analysis for all outcomes (Tables S7 and S8). The results of Cochran Q test indicated evidence of heterogeneity (Tables S9 and S10). The scatter plots of SNP-exposure and SNP-outcome associations showed the balance at around zero of the heterogeneity of genetic instruments, and the intercept from MR Egger passing through zero (Figures S1 and S3). The funnel plots appeared generally symmetrical, which suggested minimal deviation from pleiotropy (Figures S2 and S4). No outlying variants were identified from leave-one-out analysis (Figures S5 and S6). In the sensitivity analysis using genetic associations with BP unadjusted for BMI, the estimates were generally consistent in direction with the results from the main analysis (Tables S11 and S12).

Using SBP-exclusive SNPs as genetic instruments, MR analyses identified statistically significant associations of genetically proxied SBP with AF, T2DM, stroke, ischemic stroke and HF (*p* < 0.004), and a potential causal association with CAD (*p* = 0.01) (Figure 2). For a 10 mmHg increase in genetically proxied SBP, the odds ratio of AF was 1.28 (95% CI, 1.15–1.43), stroke was 1.44 (95% CI, 1.28–1.62), ischemic stroke was 1.49 (95% CI, 1.30–1.69), HF was 1.41 (95% CI, 1.20–1.65), CAD was 1.18 (95% CI, 1.03–1.36) and T2DM was 1.29 (95% CI, 1.13–1.46) (Figure 2). The MR-Egger intercepts did not detect significant directional pleiotropy (Table S13). The results from the IVW method were generally concordant in direction and magnitude with the estimates obtained using the weighted median and MR-PRESSO (Table S13, Figure S7). The sensitivity analysis after excluding SNPs that were not associated with SBP at *p*-value of 5 × 10^−8^ in the GWAS meta-analysis also gave similar MR estimates (Figure 2). MR estimates using genetic associations with BP unadjusted for BMI were generally consistent in direction with the results from the main analysis (Table S14).

Using DBP-exclusive SNPs as genetic instruments, MR analyses identified a statistically significant association of genetically proxied DBP with ischemic stroke, ischemic stroke small-vessel (*p* < 0.004) and a potential association with stroke (*p* = 0.005), CAD (*p* = 0.045) and MI (*p* = 0.05). Each genetically predicted 5 mmHg increase in DBP was associated with an OR of 1.24 in ischemic stroke (95% CI, 1.09–1.41), 1.35 in ischemic stroke small vessel (95% CI, 1.10–1.65), 1.21 in stroke (95% CI, 1.06–1.37), 1.19 in CAD (95% CI, 1.0–1.41) and 1.19 in MI (95% CI, 1.0–1.41) (Figure 3). Estimates obtained using the weighted median and MR-PRESSO were generally concordant in direction and magnitude with the results from the IVW method (Table S15, Figure S8). The MR-Egger intercepts did not detect significant directional pleiotropy (Table S15). The sensitivity analysis after excluding SNPs that were not associated with DBP at *p*-value of 5 × 10^−8^ in the GWAS meta-analysis also gave similar MR estimates (Figure 2). MR estimates using genetic associations with BP unadjusted for BMI were generally consistent in direction with the results from the main analysis (Table S16).

**Figure 4:**
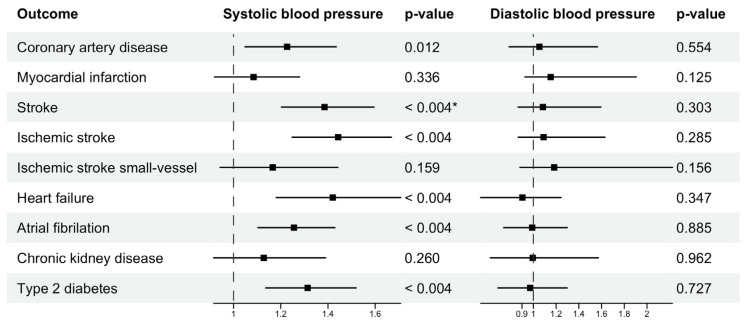
Multivariable Mendelian randomization estimates (odds ratio with 95% confidence interval per 10 mmHg increase in genetically predicted systolic blood pressure, and per 5 mmHg increase in genetically predicted diastolic blood pressure) using systolic-blood-pressure-exclusive and diastolic-blood-pressure-exclusive SNPs as genetic instruments. * *p*-value of 0.004 is the Bonferroni-corrected *p*-value in this study.

MVMR analyses were conducted using the independent SBP-exclusive SNPs and DBP-exclusive SNPs as genetic instruments. The conditional F-statistics for the genetic instruments were 39.3 for SBP and 22.8 for DBP, indicating strong instruments for MVMR. SBP was significantly associated with the increased risks of stroke, ischemic stroke, HF, AF and T2DM (*p* < 0.004), and was nominally associated with the increased risk of CAD (*p* = 0.01). After controlling for genetically predicted DBP, each genetically predicted 10 mmHg increase in SBP was associated with an OR of 1.26 in AF (95% CI, 1.10–1.43), 1.42 in HF (95% CI, 1.18–1.71), 1.39 in stroke (95% CI: 1.20–1.60), 1.44 in ischemic stroke (95% CI: 1.25–1.67), 1.31 in T2DM (95% CI, 1.14–1.52) and 1.23 in CAD (95% CI, 1.05–1.44). The effect of DBP attenuated to zero for the outcomes (Figure 4). Table 2 summarises the results from two-sample MR and MVMR analyses.

## 4. Discussion

In this study we find that genetic predisposition to higher SBP and DBP is associated with CAD, MI, stroke, HF, AF, CKD and T2DM in concordance with previous MR [15,46,47,48,49,50] and epidemiology studies [28,31]. Our novel finding is the evidence of a dichotomy between SBP and DBP on outcomes. We find CAD, stroke, ischaemic stroke and small-vessel stroke are associated with both SBP and DBP, but causal association is driven by SBP for CAD, stroke and ischaemic stroke, while it is DBP for small-vessel stroke. Furthermore, we show SBP is exclusively associated with HF, AF and T2DM. There was suggestive signals for DBP being exclusively associated with MI, but this was not statistically significant. A recent multivariable MR study using rare BP variants showed for most cardiovascular outcomes the effect of DBP was attenuated once SBP was adjusted for, but interestingly they found a putative inverse relationship between SBP and DBP on large artery stroke [51]. Our study used common GWAS SNPs for SBP and DBP and further classified these SNPs into subsets that were SBP- or DBP-exclusive or associated with both to demonstrate that SBP and DBP may be predominant causal drivers of specific cardiovascular outcomes. 

The reasons why SBP or DBP preferentially affect particular outcomes are unclear. Differences in how SBP and DBP change with age (SBP increases linearly, while DBP declines after the age of 50 years) [52] may be an explanation for any observed differential associations and indeed this has been evoked in clinical justifications for focusing on SBP in hypertension management [6]. It is also important to note that though SBP and DBP are correlated phenotypes, clinical hypertension in general tends to transition from isolated diastolic hypertension in the young through systolic-diastolic hypertension in middle-age and finally to isolated systolic hypertension in the elderly [52,53]. Several epidemiological studies indicate isolated diastolic hypertension is generally not associated with atherosclerotic cardiovascular disease, HF or CKD, independent of baseline SBP [52,53,54,55]. The J-shaped relationship between DBP and CV outcomes, specifically coronary artery disease, has been an epidemiological conundrum, but recent MR studies have refuted any non-linear relationship between DBP and CV outcomes [10,11]. Although each individual carries a preponderance of GWAS SNPs associated with both SBP and DBP, one may speculate whether the remaining SNPs that show exclusive association to either SBP or DBP may determine an individual’s predisposition to a specific CV outcome. Previous MR studies combined all BP SNPs in the construction of genetic instruments [14,49] and this may miss specific SBP or DBP effects. Previous MR studies for a causal effect of elevated BP on an increased risk of T2DM have yielded inconsistent results. In line with our study, a two-sample MR approach reported that 1 mmHg genetic increase in SBP was associated with a 2% increased risk of T2DM, by integrating summary-level GWAS data from 37,293 T2DM cases and 125,686 controls [50]. In contrast, another MR study using UKBB individual data (n = 318 664) showed that there was no clear evidence showing a causal relation from BP to T2DM risk [56]. 

The validity of MR relies on three major instrumental variable assumptions, including that the genetic instrument was robustly associated with the exposure of interest, was independent of potential confounders and was associated with the outcomes only via their association with the exposure. This study used SNPs that were strongly associated with BP traits in large GWAS studies, and the F-statistic was calculated to assess the strength of genetic instruments. All selected instruments in this MR study had F statistics > 10, suggesting that marked bias due to weak instruments is unlikely. In order to minimize the confounding bias and the pleiotropy bias, this study applied the selection criteria to select SNPs that were less likely to be pleiotropic. We also used the MR Egger intercept in MR Egger regression to detect horizontal pleiotropy effects of the instrumental variables; the evidence of horizontal pleiotropy was not detected in the analyses. Multiple sensitivity analyses using methods with different underlying assumptions (weighted median, MR Egger and MR-PRESSO) were conducted to assess the robustness of the results. The concordance in direction and magnitude of estimates across all methods indicate the credibility of the causal claim in the MR study [57]. 

Our study selected SNPs that were strongly associated with BP traits (*p*-value < 5 × 10^−8^) in large GWAS studies, using a curated database of publicly available large-scale GWAS [26,27]. We then assessed the associations of these SNPs with SBP and DBP using the summary statistics obtained from one of the largest GWAS meta-analyses for BP traits [17]. A *p*-value threshold of 5 × 10^−4^ was used to select the subsets of genetic instruments for the main analysis, and a *p*-value threshold of 5 × 10^−8^ was used for selecting instruments in the sensitivity analysis. This strategy enables the selection of some genetic variants which were significantly associated with BP traits in the large GWAS studies but did not reach the significant threshold in the GWAS meta-analyses by Evangelou et al. [17]. In total, we obtained 442 SNPs for SBP and DBP, with 25% of these SNPs being selected as genetic instruments in a recent two-sample MR study for BP traits on CVD outcomes [14]. The results in our study are generally consistent with their findings for some mutual outcomes, including MI, stroke and HF. Moreover, most of the genetic instruments (approximately 84% of the genetic instruments) in our study were associated with gene expression in at least one tissue, offering an opportunity for further functional investigations to explore causal pathways of SBP and DBP on outcomes.

Although most of the individuals in outcome GWAS were of European descent, the CARDIOGRAMplusC4D consortium included participants from multiple ancestral groups. Therefore, the confounding by population structure constitutes a possible limitation of this study. However, most of the CARDIOGRAMplusC4D consortium participants were still of European descent (77%). The other non-European studies in the consortium had been adjusted for genetic principal components to correct for population structure [19]. Another limitation of the study is the lack of correlation between the magnitude of the associations through genetic effects and the magnitude of the effect of clinical interventions, which consequently results in a difference in effect sizes between MR studies and RCTs. For this reason, MR studies are primarily utilized to assess the causal relationship of exposure on outcome [13].

## 5. Conclusions

Whilst SBP and DBP are highly correlated phenotypes and a majority of SNPs associated with BP influence both SBP and DBP, the evidence that certain SNPs influence only one of the two traits would help direct efforts using pathway and colocalisation analyses to establish SBP- and DBP-specific mechanisms. These will help expand our understanding of hypertension and its consequences and identification of druggable or actionable pathways will facilitate the transformation of hypertension management towards precision medicine.

## Figures and Tables

**Table 1:** Genome-wide association studies included in this MR study.

Traits	Consortium/Cohort	Sample Size (Cases)	Population	Reference
Systolic blood pressure	Evangelou E (2018)	757,601	European	[17]
Systolic blood pressure	Neale Lab (2017)	317,756	European	[18]
Diastolic blood pressure	Evangelou E (2018)	757,601	European	[17]
Diastolic blood pressure	Neale Lab (2017)	317,756	European	[18]
Coronary artery disease	CARDIoGRAMplusC4D	184,305 (60,801)	77% European	[19]
Myocardial infarction	CARDIoGRAMplusC4D	171,875 (43,676)	77% European	[19]
Stroke	MEGASTROKE	446,696 (40,585)	European	[20]
Heart failure	FinnGen	208,178 (13,087)	European	[21]
Atrial fibrillation	Nielsen JB (2018)	1,030,836 (60,620)	European	[22]
Chronic kidney disease	CKDGen	117,165 (12,385)	European	[23]
Type 2 diabetes	Xue A (2018)	655,666 (61,714)	European	[24]

**Table 2:** Two-sample and multivariable Mendelian randomization estimates of the effects of systolic and diastolic blood pressure on the outcomes. MR, Mendelian randomization; SBP, systolic blood pressure; DBP, diastolic blood pressure; AF, atrial fibrillation; HF, heart failure; T2DM, type 2 diabetes; CAD, coronary artery disease; MI, myocardial infarction; CKD, chronic kidney disease. * SBP-exclusive SNPs. ** DBP-exclusive SNPs. § SBP-excusive SNPs and DBP-exclusive SNPs.

	Two-Sample MR				Multivariable MR			
	SBP_Excl (SBP) *		DBP_Excl (DBP) **		SBPexc+DBPexc (SBP) §		SBPexc+DBPexc (DBP) §	
	Estimates	*p*-Value	Estimates	*p*-Value	Estimate	*p*-Value	Estimate	*p*-Value
AF	0.0247	8.11 × 10^−6^	0.0104	0.3872	0.0244	0.0007	−0.0347	0.8853
HF	0.0341	2.17 × 10^−5^	−0.003	0.8529	0.0516	0.0003	−0.1297	0.3470
T2DM	0.0252	0.0001	0.0162	0.3297	0.0260	0.0003	−0.0151	0.7266
CAD	0.0172	0.0148	0.0347	0.0453	0.0238	0.0116	−0.0162	0.5542
Stroke	0.0365	3.03 × 10^−9^	0.0375	0.0047	0.0398	1.17 × 10^−5^	0.0195	0.3034
Ischemic stroke	0.0396	4.07 × 10^−9^	0.0431	0.0009	0.0418	1.85 × 10^−6^	0.0250	0.2854
Ischemic stroke small-vessel	0.0181	0.0553	0.06	0.0034	0.0108	0.1585	0.0484	0.1559
MI	0.0079	0.2816	0.0341	0.0520	0.0117	0.3364	0.0096	0.1249
CKD	0.0111	0.2045	0.0118	0.6163	0.0219	0.2598	−0.0226	0.9621

## Data Availability

Data used in this study were obtained from online resources which are available to all researchers–PhenoScanner (http://www.phenoscanner.medschl.cam.ac.uk/ accessed on 20 June 2022), GWAS Catalog (https://www.ebi.ac.uk/gwas/ accessed on 20 June 2022), MR-Base (https://www.mrbase.org accessed on 20 June 2022).

## Supplementary Materials

The following are available online at https://www.mdpi.com/article/10.3390/genes13071226/s1, Supplementary Checklist: STROBE-MR checklist of recommended items to address in reports of Mendelian randomization studies; Figure S1: Scatter plots for MR analyses of the causal effect of systolic blood pressure on (a) atrial fibrillation, (b) coronary artery disease, (c) myocardial infarction, (d) stroke, (e) ischemic stroke, (f) ischemic stroke (small-vessel), (g) heart failure, (h) chronic kidney disease, (i) type 2 diabetes, using inverse-variance weighted, weighted median, and MR Egger methods; Figure S2: MR-Egger regression funnel plots for systolic blood pressure on (a) atrial fibrillation, (b) coronary artery disease, (c) myocardial infarction, (d) stroke, (e) ischemic stroke, (f) ischemic stroke (small-vessel), (g) heart failure, (h) chronic kidney disease, (i) type 2 diabetes; Figure S3. Scatter plots for MR analyses of the causal effect of diastolic blood pressure on (a) atrial fibrillation, (b) coronary artery disease, (c) myocardial infarction, (d) stroke, (e) ischemic stroke, (f) ischemic stroke (small-vessel), (g) heart failure, (h) chronic kidney disease, (i) type 2 diabetes, using inverse-variance weighted, weighted median, and MR Egger methods; Figure S4: MR-Egger regression funnel plots for diastolic blood pressure on (a) atrial fibrillation, (b) coronary artery disease, (c) myocardial infarction, (d) stroke, (e) ischemic stroke, (f) ischemic stroke (small-vessel), (g) heart failure, (h) chronic kidney disease, (i) type 2 diabetes; Figure S5: Leave-one-out plots for MR analyses of the causal effect of systolic blood pressure on (a) atrial fibrillation, (b) coronary artery disease, (c) myocardial infarction, (d) stroke, (e) ischemic stroke, (f) ischemic stroke (small-vessel), (g) heart failure, (h) chronic kidney disease, (i) type 2 diabetes, using inverse-variance weighted, weighted median, and MR Egger methods; Figure S6: Leave-one-out plots for MR analyses of the causal effect of diastolic blood pressure on (a) atrial fibrillation, (b) coronary artery disease, (c) myocardial infarction, (d) stroke, (e) ischemic stroke, (f) ischemic stroke (small-vessel), (g) heart failure, (h) chronic kidney disease, (i) type 2 diabetes, using inverse-variance weighted, weighted median, and MR Egger methods; Figure S7: Scatter plots for MR analyses of the independent causal effect of systolic blood pressure on (a) atrial fibrillation, (b) coronary artery disease, (c) heart failure, (d) stroke, (e) ischemic stroke, (f) ischemic stroke (small-vessel), (g) type 2 diabetes, using inverse-variance weighted, weighted median, and MR Egger methods; Figure S8: Scatter plots for MR analyses of the independent causal effect of diastolic blood pressure on (a) myocardial infarction, (b) stroke, (c) ischemic stroke, (d) ischemic stroke (small-vessel) using inverse-variance weighted, weighted median, and MR Egger methods; Figure S9: MR-Egger regression funnel plots for systolic blood pressure on (a) atrial fibrillation, (b) coronary artery disease, (c) heart failure, (d) stroke, (e) ischemic stroke, (f) ischemic stroke (small-vessel), (g) type 2 diabetes, using systolic blood pressure-exclusive SNPs; Figure S10: MR-Egger regression funnel plots for diastolic blood pressure on (a) myocardial infarction, (b) stroke, (c) ischemic stroke, (d) ischemic stroke (small-vessel), using diastolic blood pressure-exclusive SNPs; Table S1: SBP-and-DBP-associated SNPs included in the Mendelian randomisation analysis for SBP; Table S2: SBP-and-DBP-associated SNPs included in the Mendelian randomisation analysis for DBP; Table S3: SBP-exclusive SNPs included in the Mendelian randomization analysis for SBP; Table S4: DBP-exclusive SNPs included in the Mendelian randomization analysis for DBP; Table S5. SBP-exclusive SNPs included in the sensitivity analysis for SBP; Table S6. DBP-exclusive SNPs included in the sensitivity analysis for DBP; Table S7: Mendelian randomization estimates of systolic blood pressure (per 10 mmHg) on cardiovascular outcomes using SBP-and-DBP-associated SNPs; Table S8: Mendelian randomization estimates of diastolic blood pressure (per 5 mmHg) on cardiovascular outcomes using SBP-and-DBP-associated SNPs; Table S9: Cochran’s Q test results in 2-sample Mendelian randomisation analyses of systolic blood pressure on outcomes using SNPs associated with both systolic blood pressure and diastolic blood pressure as genetic instruments; Table S10: Cochran’s Q test results in 2-sample Mendelian randomisation analyses of diastolic blood pressure on outcomes using SNPs associated with both systolic blood pressure and diastolic blood pressure as genetic instruments; Table S11: Mendelian randomization estimates of systolic blood pressure (per SD increment) on cardiovascular outcomes using genetic associations with blood pressure unadjusted for body mass index (using SBP+DBP SNPs as genetic instruments); Table S12: Mendelian randomization estimates of diastolic blood pressure (per SD increment) on cardiovascular outcomes using genetic associations with blood pressure unadjusted for body mass index (using SBP+DBP SNPs as genetic instruments); Table S13: Mendelian randomization estimates of systolic blood pressure (per 10 mmHg) on cardiovascular outcomes using SBP-exclusive SNPs; Table S14: Mendelian randomization estimates of systolic blood pressure (per SD increment) on cardiovascular outcomes using genetic associations with blood pressure unadjusted for body mass index (using SBP-exclusive SNPs as genetic instruments); Table S15: Mendelian randomization estimates of diastolic blood pressure (per 5 mmHg) on cardiovascular outcomes using DBP-exclusive SNPs; Table S16: Mendelian randomization estimates of diastolic blood pressure (per SD increment) on cardiovascular outcomes using genetic associations with blood pressure unadjusted for body mass index (using DBP-exclusive SNPs as genetic instruments).
