# Supplementary material for: Unravelling the Distinct Effects of Systolic and Diastolic Blood Pressure Using Mendelian Randomisation
Source: Genes (Basel). 2022 Jul 9;13(7):1226. doi: 10.3390/genes13071226 (PMC9323763; doi:10.3390/genes13071226)
Supplement: Supplementary file 1 [file genes-13-01226-s001.zip › Data Supplement Figures.pdf]

Supplementary Checklist – STROBE-MR checklist of recommended items to address in reports of Mendelian randomization studies (1)

1. TITLE and ABSTRACT
Indicate Mendelian randomization as the study's design in the title and/or the abstract.
Detailed in the Title and Abstract sections.

INTRODUCTION
2. Background
Explain the scientific background and rationale for the reported study. Is causality between exposure and outcome plausible? Justify why MR is a helpful method to address the study question.
Detailed in the Background section.

3. Objectives
State specific objectives clearly, including pre-specified causal hypotheses (if any).
Detailed in the Background section.

METHODS
4. Study design and data sources
Present key elements of study design early in the paper. Consider including a table listing sources of data for all phases of the study. For each data source contributing to the analysis, describe the following:
a) Describe the study design and the underlying population from which it was drawn. Describe also the setting, locations, and relevant dates, including periods of recruitment, exposure, follow-up, and data collection, if available.
b) Give the eligibility criteria, and the sources and methods of selection of participants.
c) Explain how the analyzed sample size was arrived at.
d) Describe measurement, quality and selection of genetic variants.
e) For each exposure, outcome and other relevant variables, describe methods of assessment and, in the case of diseases, the diagnostic criteria used.
f) Provide details of ethics committee approval and participant informed consent, if relevant.
Detailed in the Methods section.

5. Assumptions
Explicitly state assumptions for the main analysis (e.g. relevance, exclusion, independence, homogeneity) as well assumptions for any additional or sensitivity analysis.
Detailed in the Methods section.

6. Statistical methods: main analysis
Describe statistical methods and statistics used.
a) Describe how quantitative variables were handled in the analyses (i.e., scale, units, model).
b) Describe the process for identifying genetic variants and weights to be included in the analyses (i.e, independence and model). Consider a flow diagram.
c) Describe the MR estimator, e.g. two-stage least squares, Wald ratio, and related statistics. Detail the included covariates and, in case of two-sample MR, whether the same covariate set was used for adjustment in the two samples.
d) Explain how missing data were addressed.
e) If applicable, say how multiple testing was dealt with.
Detailed in the Methods section.

7. Assessment of assumptions
Describe any methods used to assess the assumptions or justify their validity.
Detailed in the Methods, and Discussion sections.

8. Sensitivity analyses
Describe any sensitivity analyses or additional analyses performed.
Detailed in the Methods sections.

9. Software and pre-registration
a) Name statistical software and package(s), including version and settings used.
Detailed in the Methods sections.

b) State whether the study protocol and details were pre-registered (as well as when and where).
Detailed in the Methods sections.

RESULTS
10. Descriptive data
a) Report the numbers of individuals at each stage of included studies and reasons for exclusion. Consider use of a flow-diagram.
b) Report summary statistics for phenotypic exposure(s), outcome(s) and other relevant variables (e.g. means, standard deviations, proportions).
c) If the data sources include meta-analyses of previous studies, provide the number of studies, their reported ancestry, if available, and assessments of heterogeneity across these studies. Consider using a supplementary table for each data source.
d) For two-sample Mendelian randomization:
i. Provide information on the similarity of the genetic variant-exposure associations between the exposure and outcome samples.
ii. Provide information on extent of sample overlap between the exposure and outcome data sources.
Detailed in the Methods, and Results sections.

11. Main results
a) Report the associations between genetic variant and exposure, and between genetic variant and outcome, preferably on an interpretable scale (e.g. comparing 25th and 75th percentile of allele count or genetic risk score, if individual-level data available).
b) Report causal effect estimate between exposure and outcome, and the measures of uncertainty from the MR analysis. Use an intuitive scale, such as odds ratio, or relative risk, per standard deviation difference.
c) If relevant, consider translating estimates of relative risk into absolute risk for a meaningful time-period.
d) Consider any plots to visualize results (e.g. forest plot, scatterplot of associations between genetic variants and outcome versus between genetic variants and exposure).
Detailed in the Results, Supplementary Tables and Supplementary Figures sections.

12. Assessment of assumptions
a) Assess the validity of the assumptions.
b) Report any additional statistics (e.g., assessments of heterogeneity, such as I2, Q statistic).
Detailed in the Results, Supplementary Tables and Discussion sections.

13. Sensitivity and additional analyses

a) Use sensitivity analyses to assess the robustness of the main results to violations of the assumptions.
b) Report results from other sensitivity analyses (e.g., replication study with different dataset, analyses of subgroups, validation of instrument(s), simulations, etc.).
c) Report any assessment of direction of causality (e.g., bidirectional MR).
d) When relevant, report and compare with estimates from non-MR analyses.
e) Consider any additional plots to visualize results (e.g., leave-one-out analyses).
Detailed in the Results, Supplementary Tables and Supplementary Figures sections.

DISCUSSION
14. Key results
Summarize key results with reference to study objectives.
Detailed in the Discussion section.

15. Limitations
Discuss limitations of the study, taking into account the validity of the MR assumptions, other sources of potential bias, and imprecision. Discuss both direction and magnitude of any potential bias, and any efforts to address them.
Detailed in the Discussion section.

16. Interpretation
a) Give a cautious overall interpretation of results considering objectives and limitations. Compare with results from other relevant studies.
b) Discuss underlying biological mechanisms that could be modelled by using the genetic variants to assess the relationship between the exposure and the outcome.
c) Discuss whether the results have clinical or policy relevance, and whether interventions could have the same size effect.
Detailed in the Discussion section.

17. Generalizability
Discuss the generalizability of the study results (a) to other populations (i.e. external validity), (b) across other exposure periods/timings, and (c) across other levels of exposure.
Detailed in the Discussion section.

OTHER INFORMATION
18. Funding
Give the source of funding and the role of the funders for the present study and, if applicable, for the original study or studies on which the present article is based.
Detailed in the Funding section.

19. Data and data sharing
Present data used to perform all analyses or report where and how the data can be accessed. State whether statistical code is publicly accessible and if so, where.
Detailed in the Methods section.

20. Conflicts of Interest
All authors should declare all potential conflicts of interest.
Detailed in the Conflicts of Interest section.

1. Skrivankova VW, Richmond RC, Woolf BAR, Davies NM, Swanson SA, VanderWeele TJ, et al. Strengthening the reporting of observational studies in epidemiology using mendelian randomisation (STROBE-MR): explanation and elaboration. BMJ. 2021;375:n2233.

Supplementary Figures

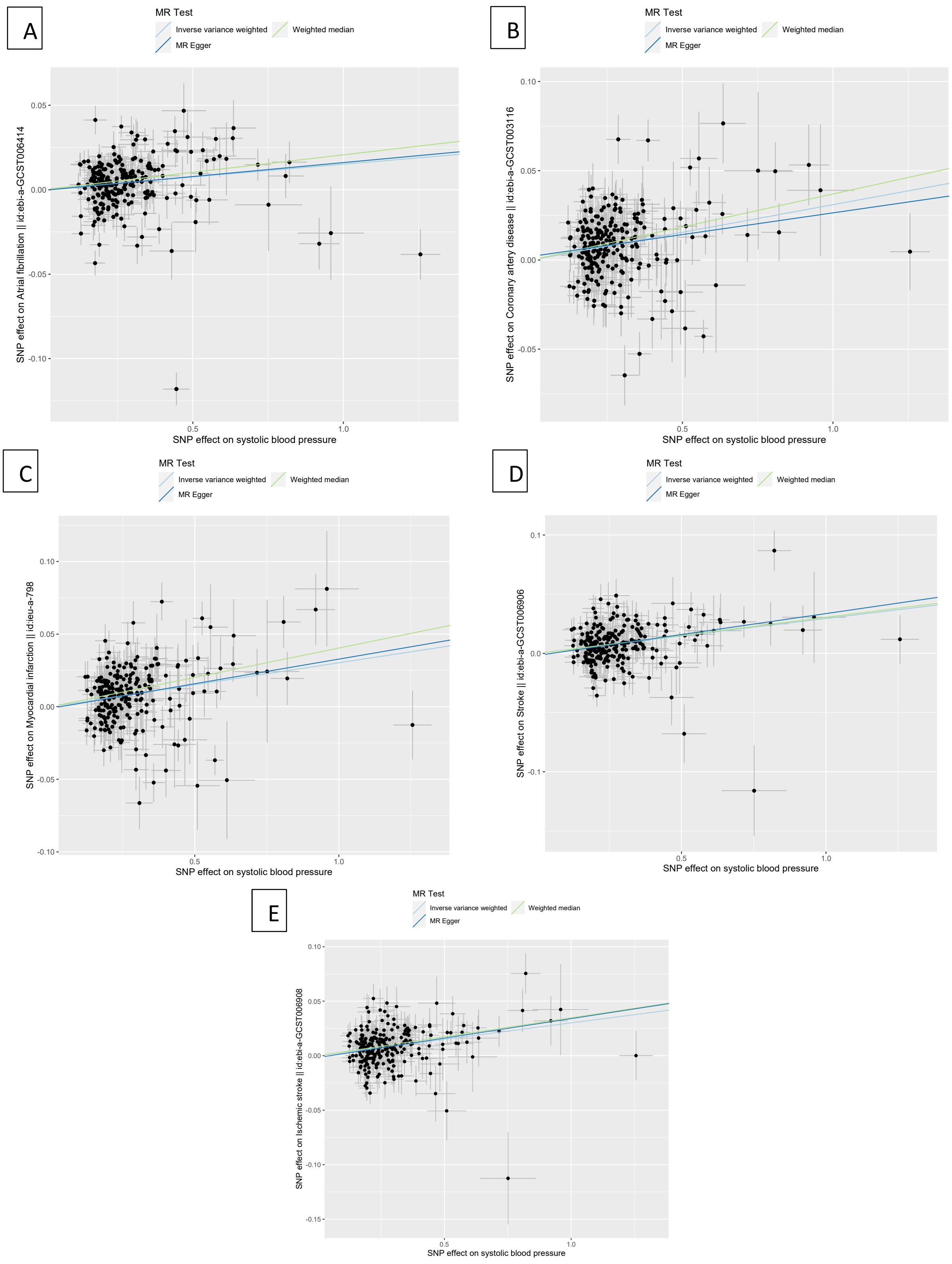

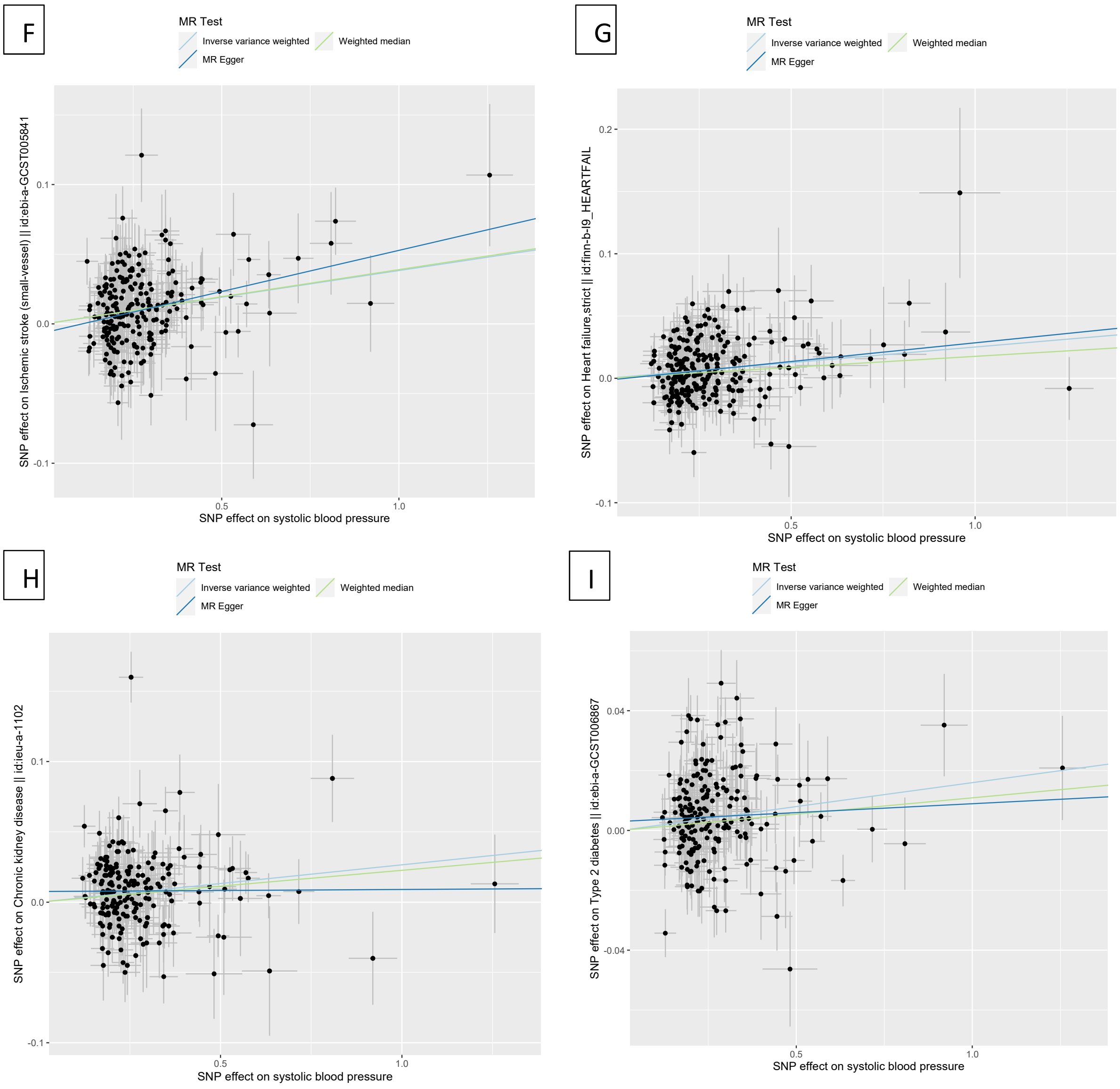

Figure S1. Scatter plots for MR analyses of the causal effect of systolic blood pressure on (A) atrial fibrillation, (B) coronary artery disease, (C) myocardial infarction, (D) stroke, (E) ischemic stroke, (F) ischemic stroke (small-vessel), (G) heart failure, (H) chronic kidney disease, (I) type 2 diabetes, using inverse-variance weighted, weighted median, and MR Egger methods. SNP = single nucleotide polymorphism, MR = Mendelian randomization.

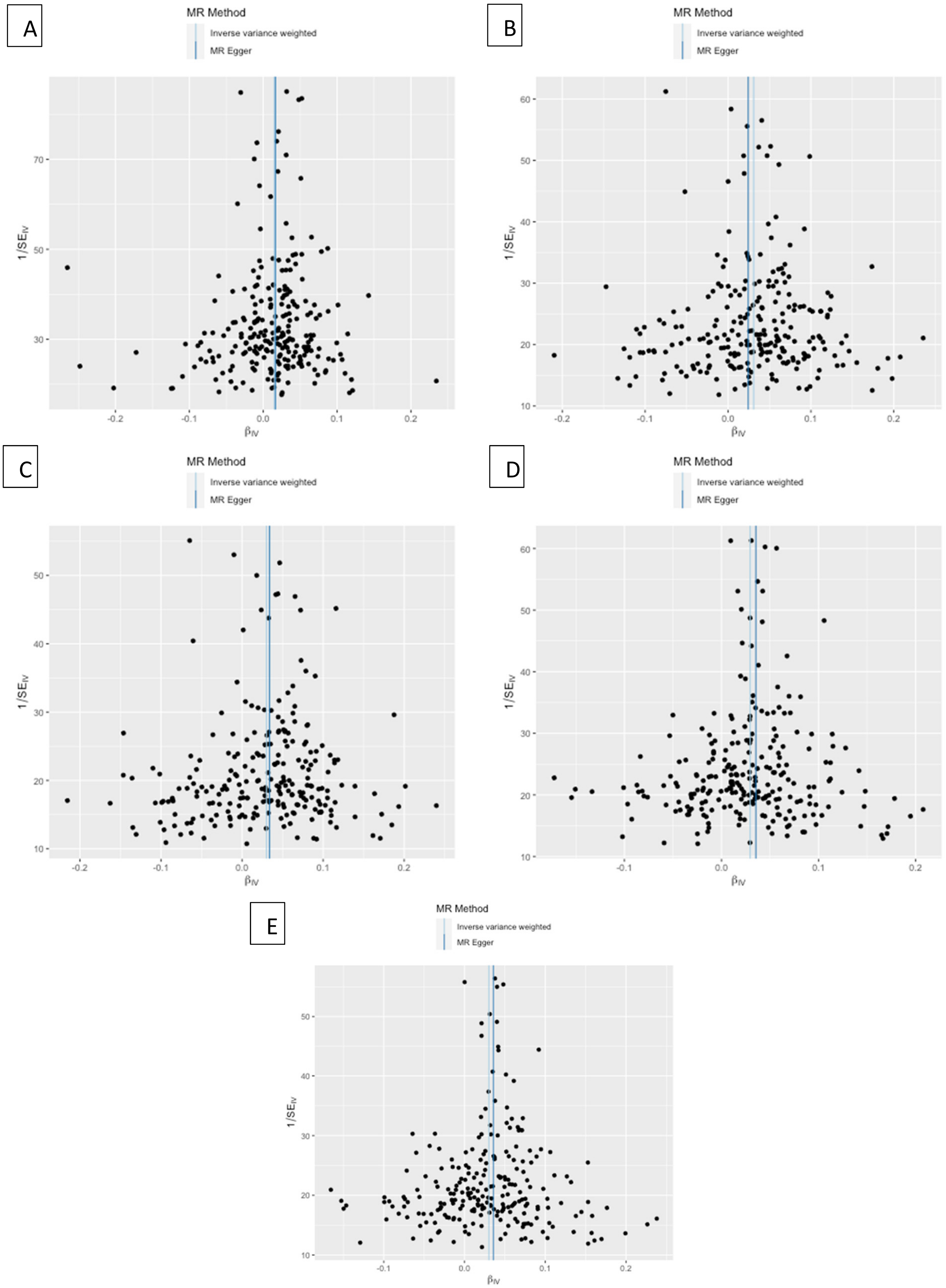
A
MR Method
Inverse variance weighted
MR Egger
1/SE_IV
β_IV
-0.2
-0.1
0.0
0.1
0.2
30
50
70
B
MR Method
Inverse variance weighted
MR Egger
1/SE_IV
β_IV
10
20
30
40
50
60
C
MR Method
Inverse variance weighted
MR Egger
1/SE_IV
β_IV
D
MR Method
Inverse variance weighted
MR Egger
1/SE_IV
β_IV
E
MR Method
Inverse variance weighted
MR Egger
1/SE_IV
β_IV

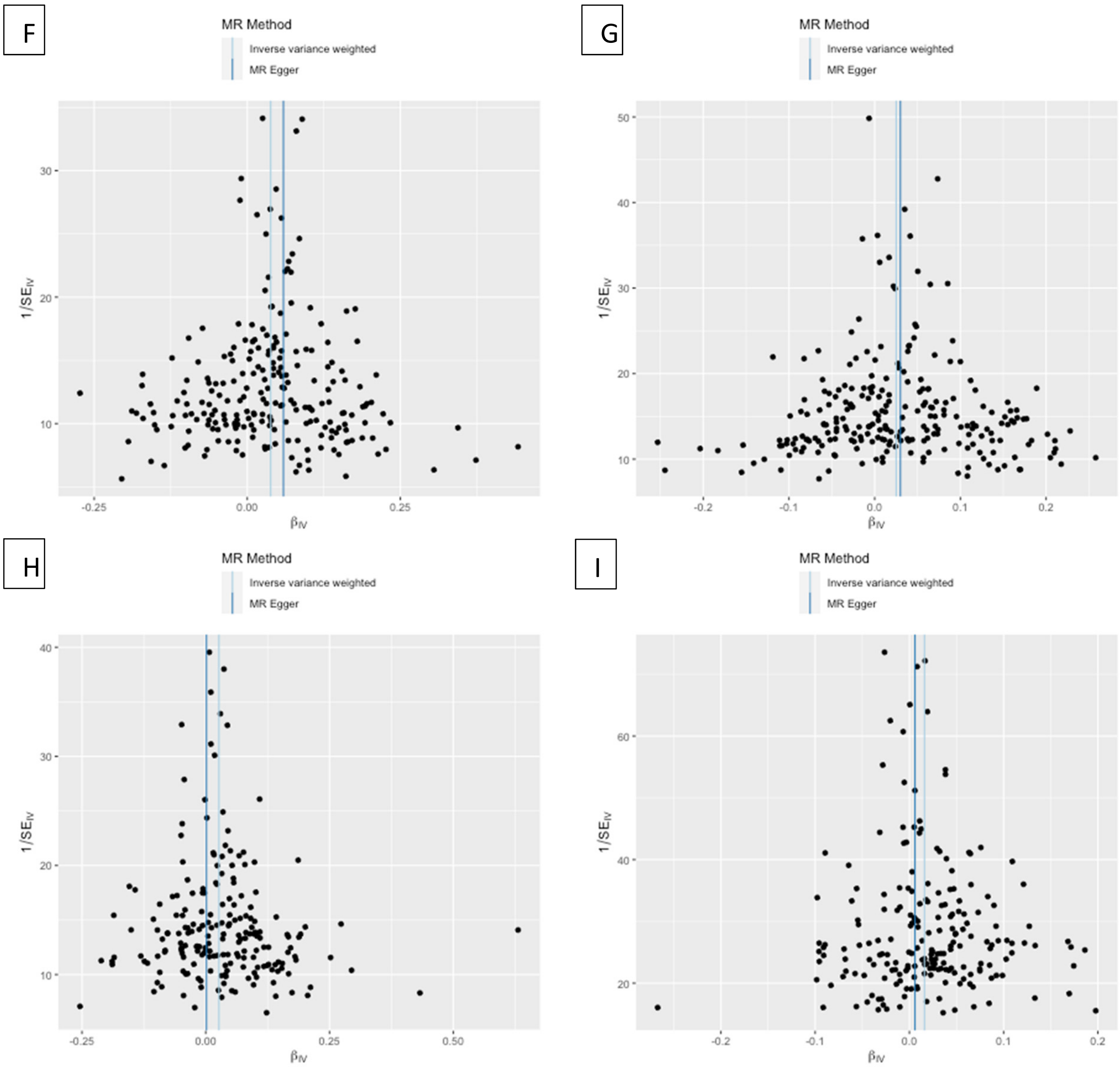

Figure S2. MR-Egger regression funnel plots for systolic blood pressure on (A) atrial fibrillation, (B) coronary artery disease, (C) myocardial infarction, (D) stroke, (E) ischemic stroke, (F) ischemic stroke (small-vessel), (G) heart failure, (H) chronic kidney disease, (I) type 2 diabetes. IV = instrumental variable; MR = Mendelian randomization; SE = standard error.

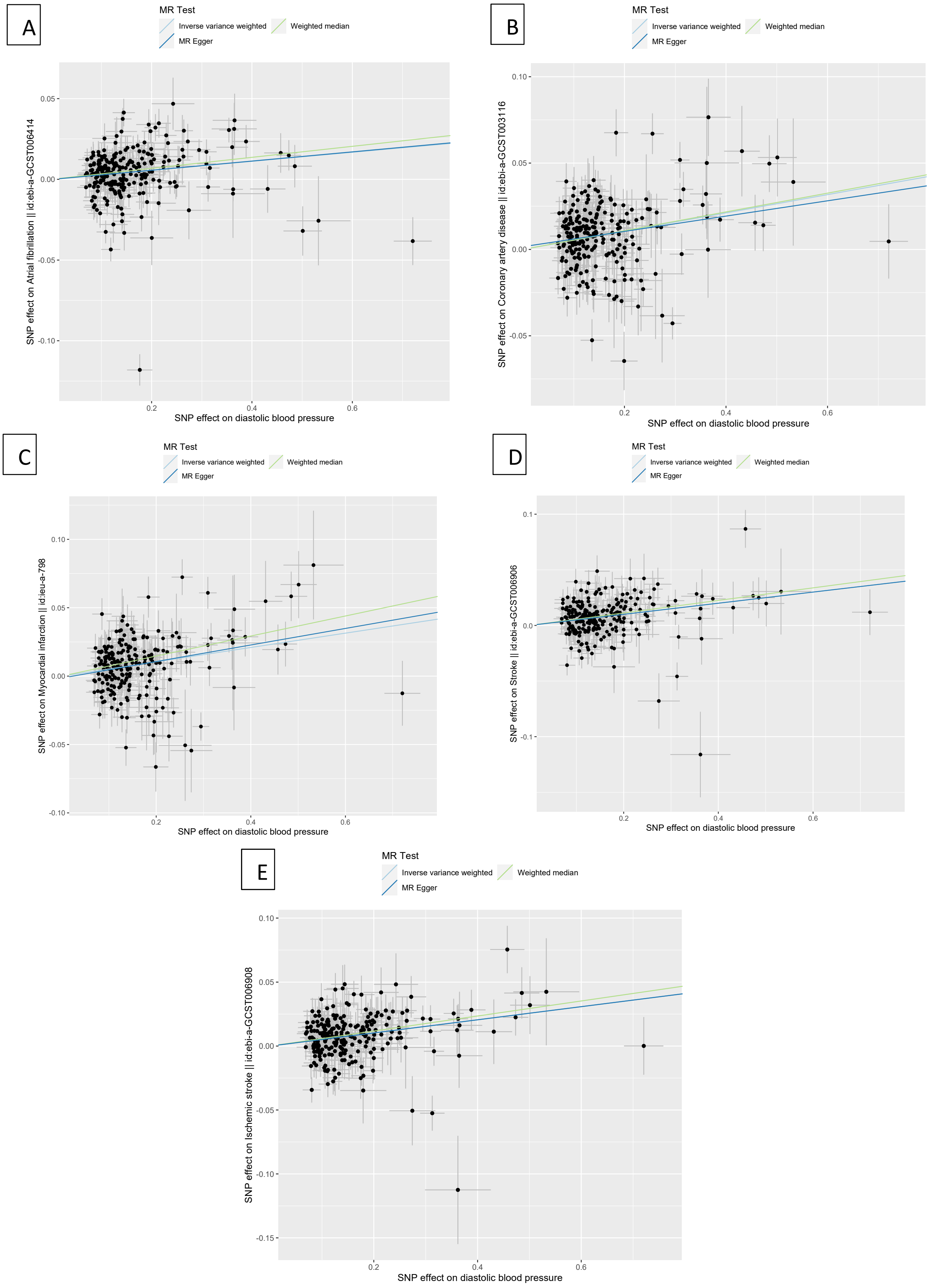
A
MR Test
Inverse variance weighted
Weighted median
MR Egger
SNP effect on Atrial fibrillation || id:ebi-a-GCST006414
SNP effect on diastolic blood pressure
B
MR Test
Inverse variance weighted
Weighted median
MR Egger
SNP effect on Coronary artery disease || id:ebi-a-GCST003116
SNP effect on diastolic blood pressure
C
MR Test
Inverse variance weighted
Weighted median
MR Egger
SNP effect on Myocardial infarction || id:ieu-a-798
SNP effect on diastolic blood pressure
D
MR Test
Inverse variance weighted
Weighted median
MR Egger
SNP effect on Stroke || id:ebi-a-GCST006906
SNP effect on diastolic blood pressure
E
MR Test
Inverse variance weighted
Weighted median
MR Egger
SNP effect on Ischemic stroke || id:ebi-a-GCST006908
SNP effect on diastolic blood pressure

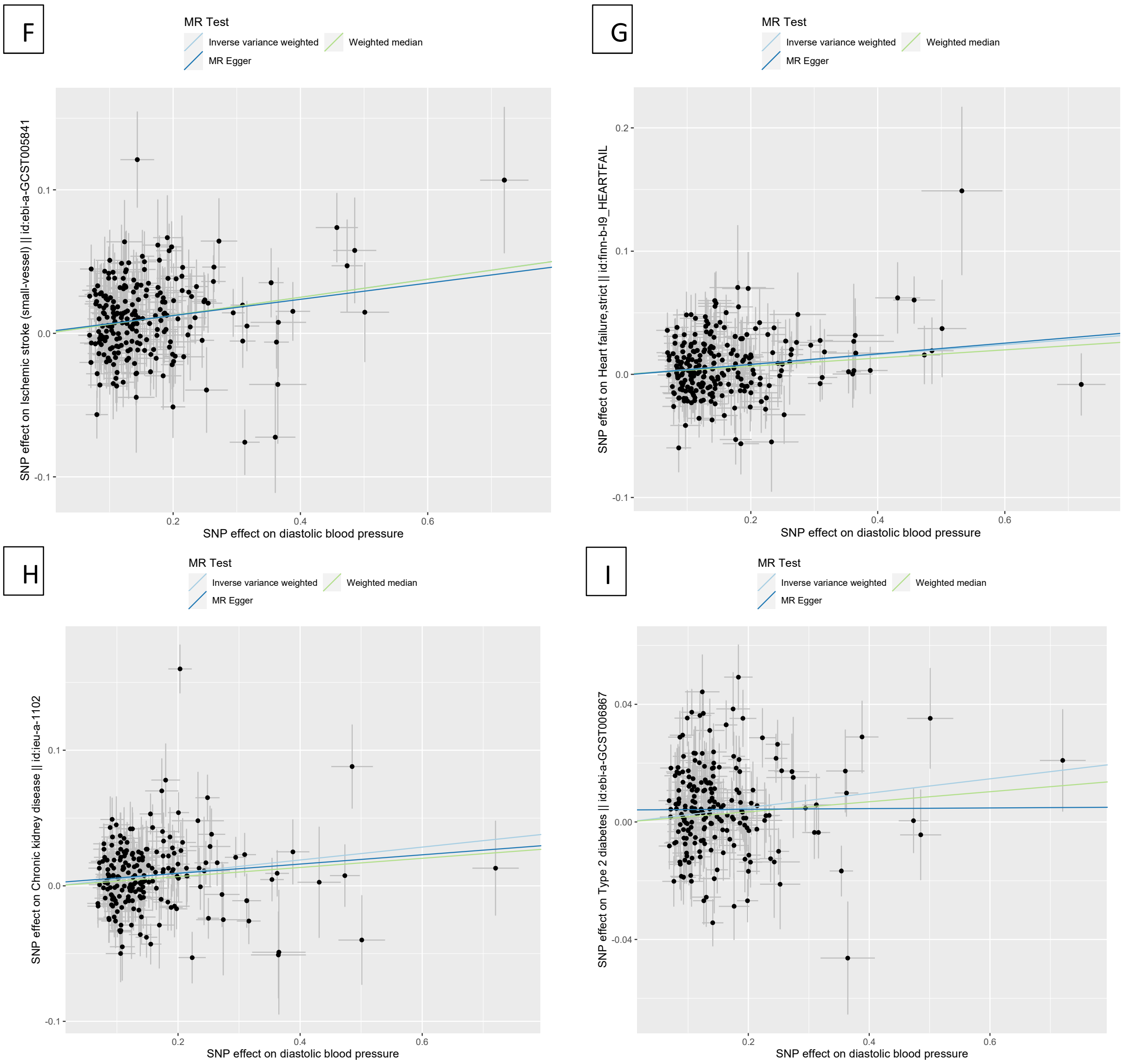

Figure S3. Scatter plots for MR analyses of the causal effect of diastolic blood pressure on (A) atrial fibrillation, (B) coronary artery disease, (C) myocardial infarction, (D) stroke, (E) ischemic stroke, (F) ischemic stroke (small-vessel), (G) heart failure, (H) chronic kidney disease, (I) type 2 diabetes, using inverse-variance weighted, weighted median, and MR Egger methods. SNP = single nucleotide polymorphism, MR = Mendelian randomization.

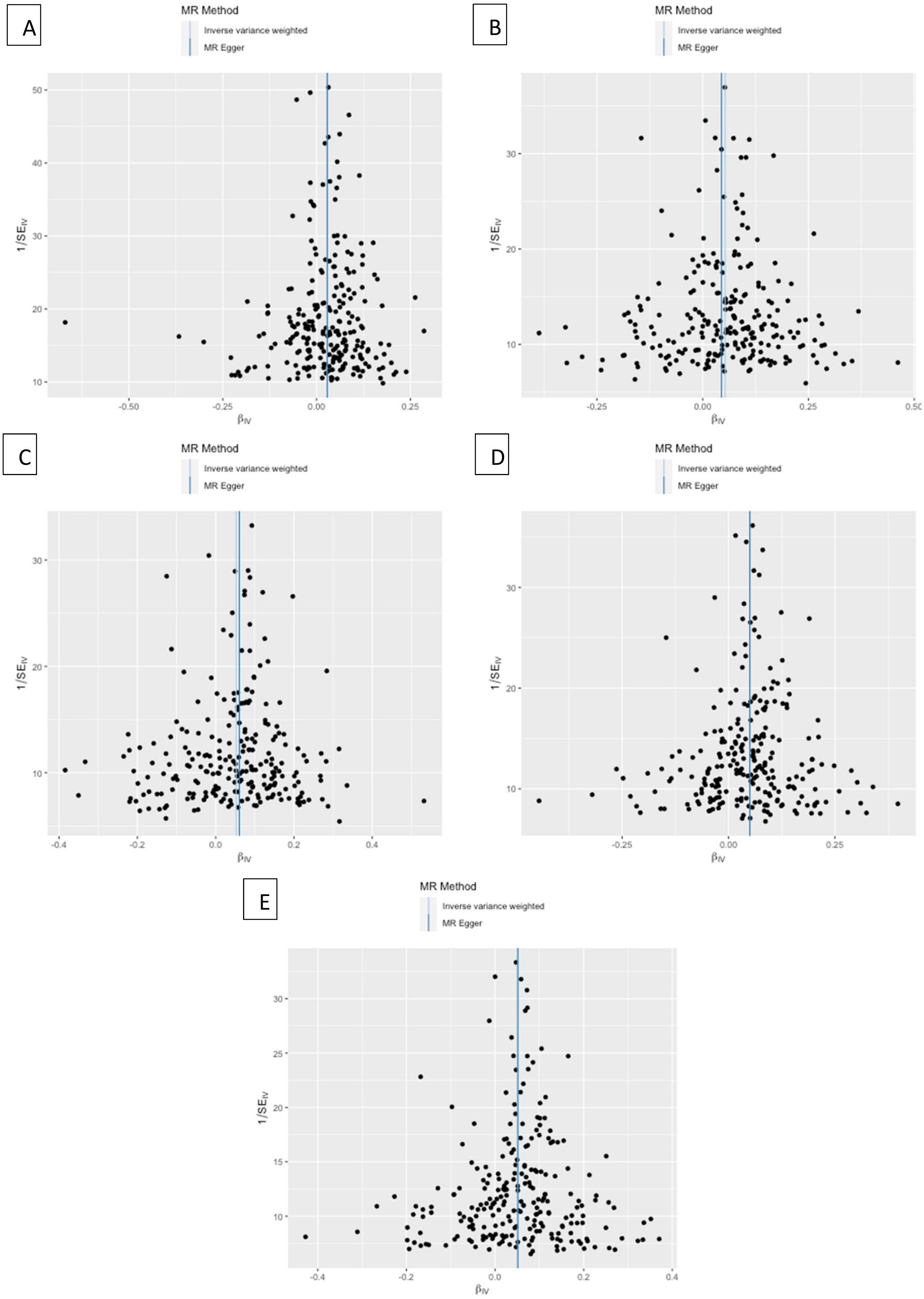
A
B
C
D
E
MR Method
Inverse variance weighted
MR Egger
1/SE_IV
β_IV

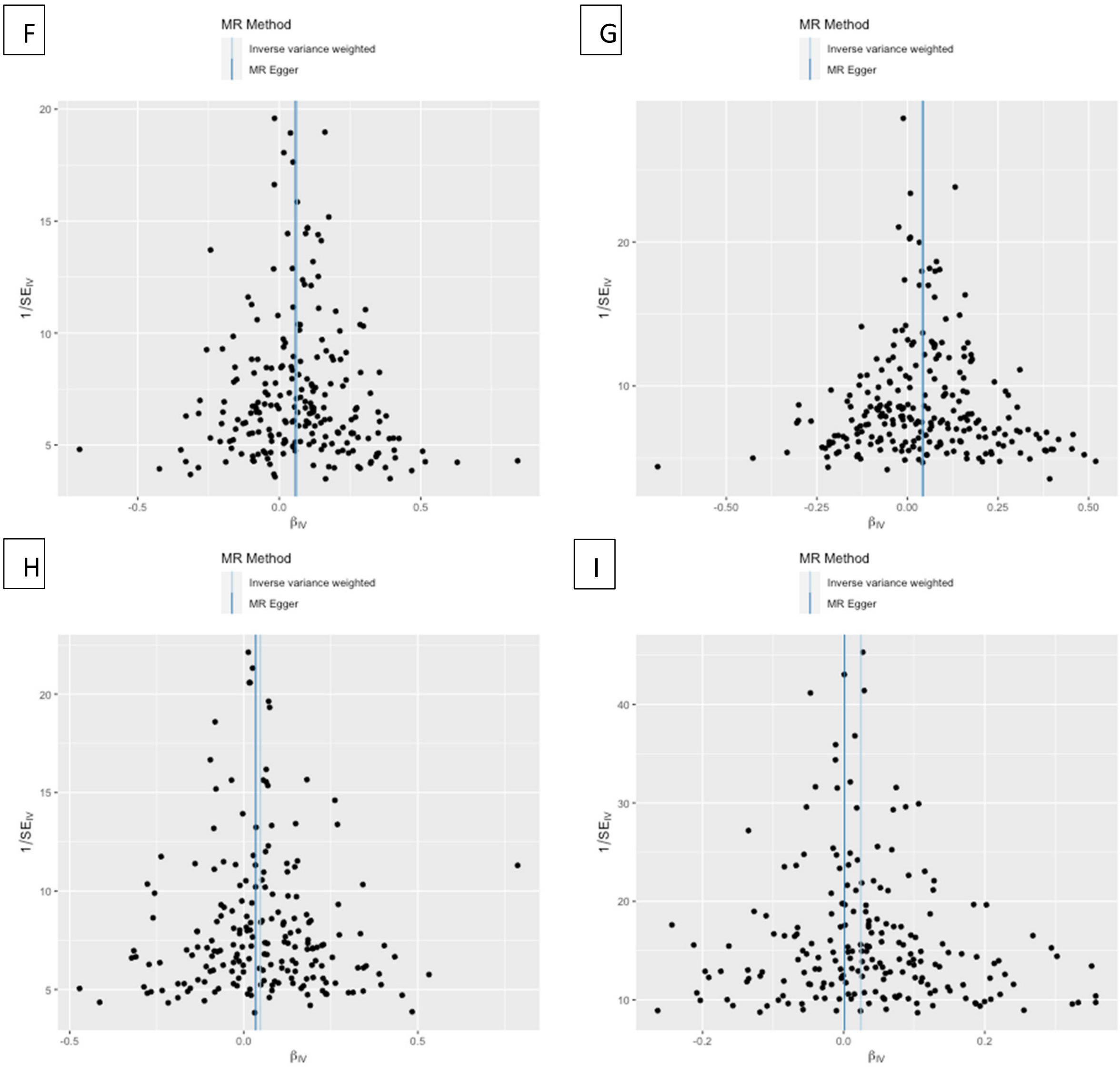

Figure S4. MR-Egger regression funnel plots for diastolic blood pressure on (A) atrial fibrillation, (B) coronary artery disease, (C) myocardial infarction, (D) stroke, (E) ischemic stroke, (F) ischemic stroke (small-vessel), (G) heart failure, (H) chronic kidney disease, (I) type 2 diabetes. IV = instrumental variable; MR = Mendelian randomization; SE = standard error.

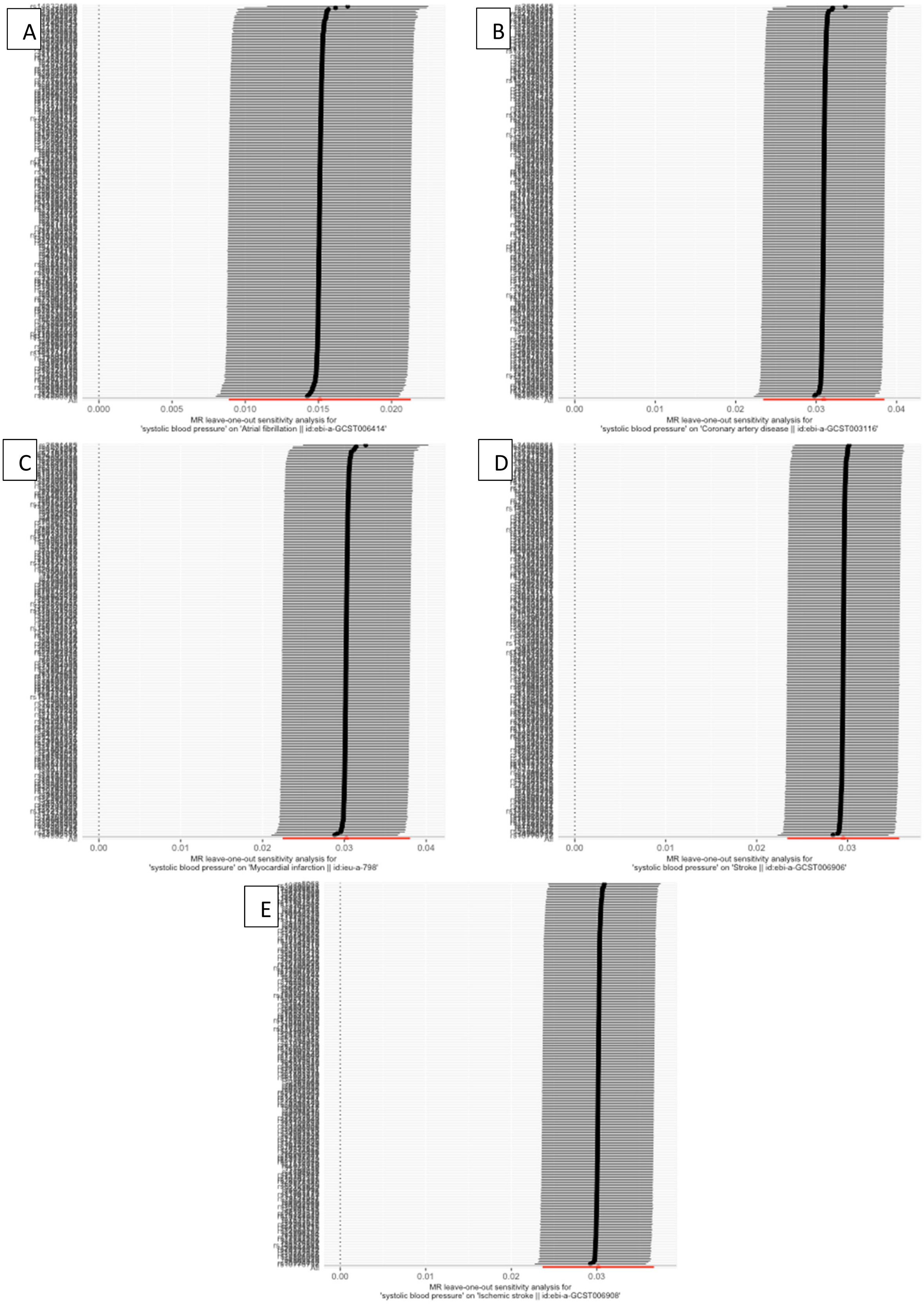
A
B
C
D
E
0.000
0.005
0.010
0.015
0.020
MR leave-one-out sensitivity analysis for
'systolic blood pressure' on 'Atrial fibrillation || id:ebi-a-GCST006414'
0.00
0.01
0.02
0.03
0.04
MR leave-one-out sensitivity analysis for
'systolic blood pressure' on 'Coronary artery disease || id:ebi-a-GCST003116'
MR leave-one-out sensitivity analysis for
'systolic blood pressure' on 'Myocardial infarction || id:ieu-a-798'
MR leave-one-out sensitivity analysis for
'systolic blood pressure' on 'Stroke || id:ebi-a-GCST006906'
MR leave-one-out sensitivity analysis for
'systolic blood pressure' on 'Ischemic stroke || id:ebi-a-GCST006908'
All

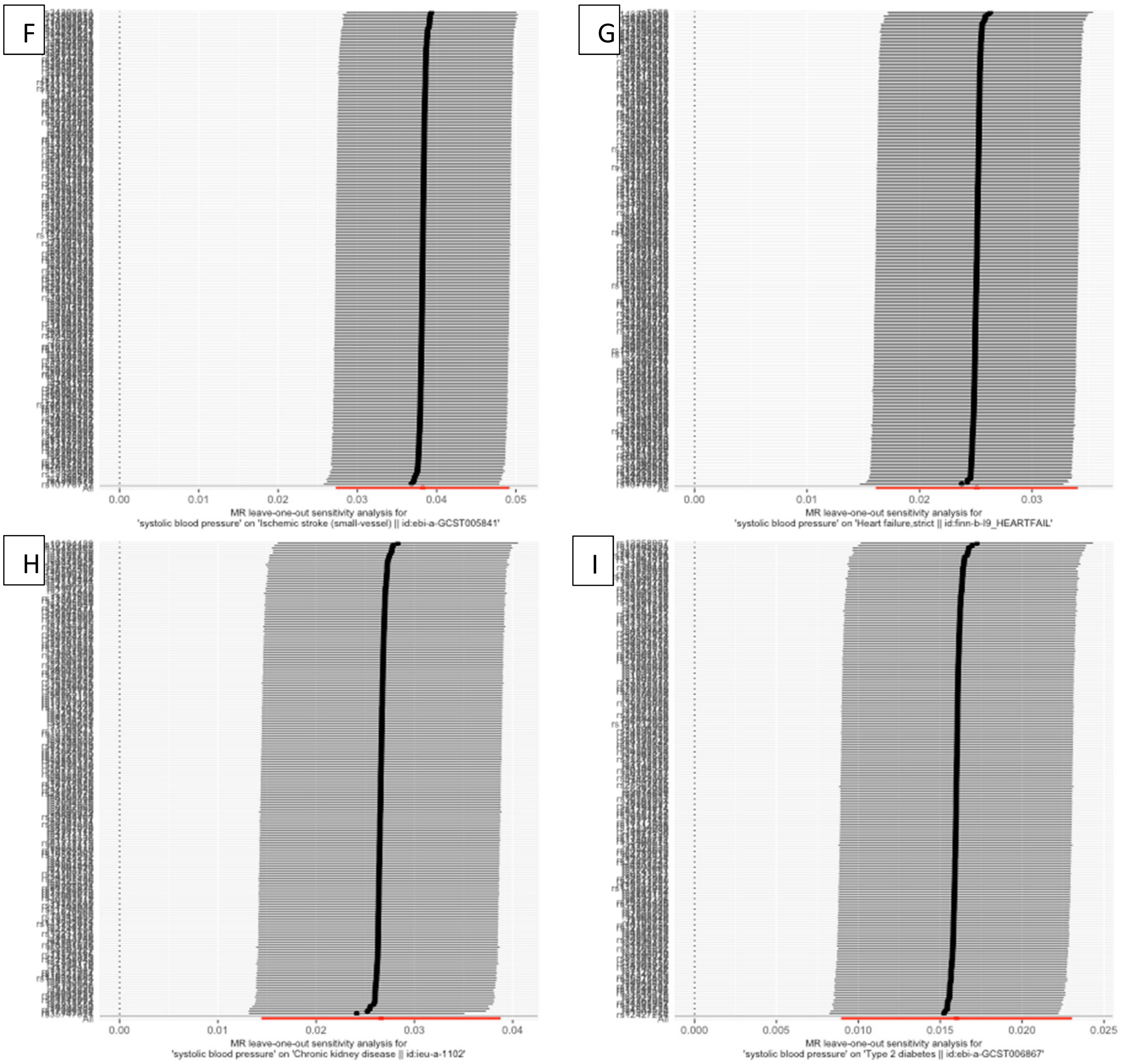

Figure S5. Leave-one-out plots for MR analyses of the causal effect of systolic blood pressure on (A) atrial fibrillation, (B) coronary artery disease, (C) myocardial infarction, (D) stroke, (E) ischemic stroke, (F) ischemic stroke (small-vessel), (G) heart failure, (H) chronic kidney disease, (I) type 2 diabetes, using inverse-variance weighted, weighted median, and MR Egger methods. SNP = single nucleotide polymorphism, MR = Mendelian randomization.

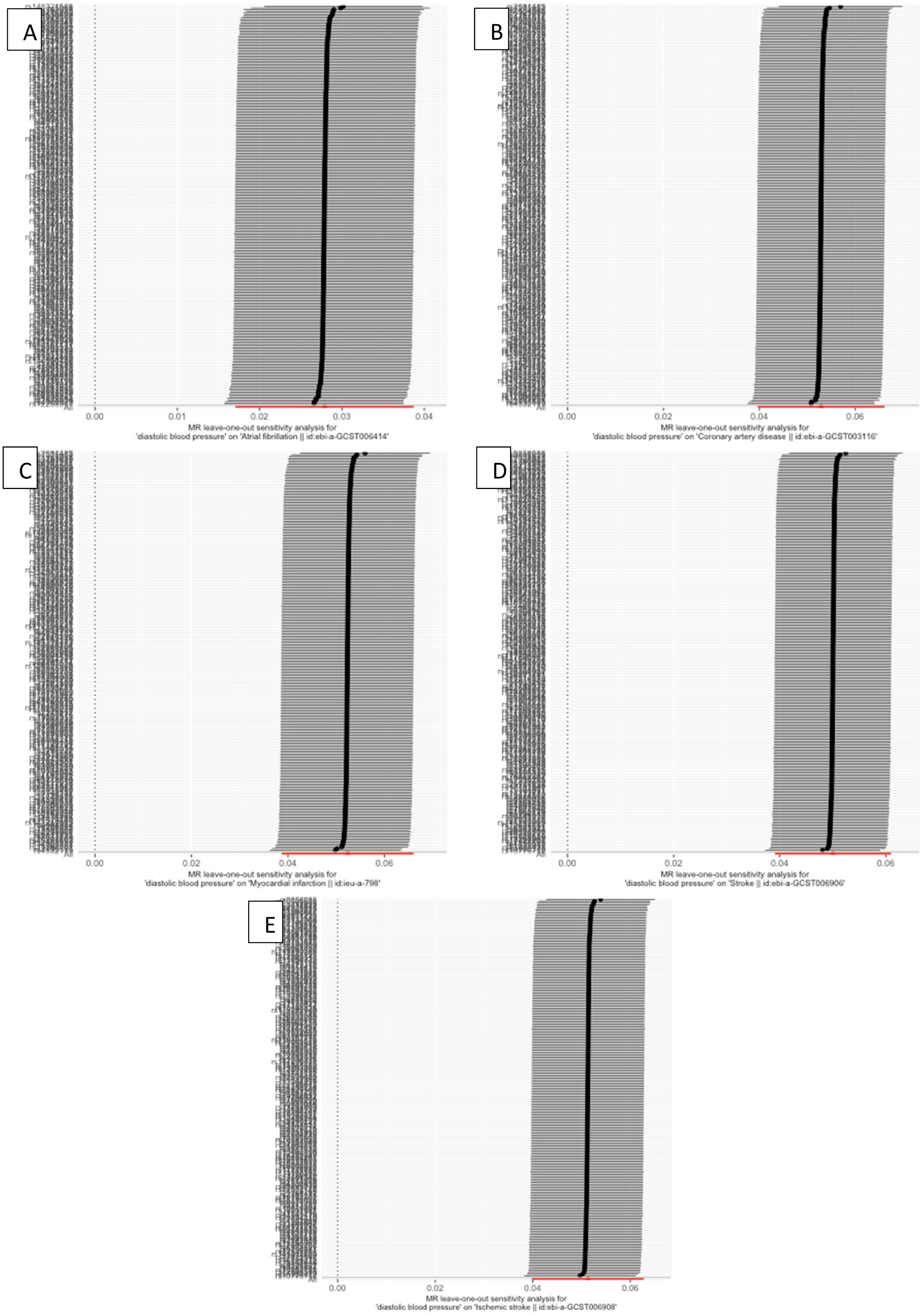
A
MR leave-one-out sensitivity analysis for
'diastolic blood pressure' on 'Atrial fibrillation || id:ebi-a-GCST006414'
B
MR leave-one-out sensitivity analysis for
'diastolic blood pressure' on 'Coronary artery disease || id:ebi-a-GCST003116'
C
MR leave-one-out sensitivity analysis for
'diastolic blood pressure' on 'Myocardial infarction || id:ieu-a-798'
D
MR leave-one-out sensitivity analysis for
'diastolic blood pressure' on 'Stroke || id:ebi-a-GCST006906'
E
MR leave-one-out sensitivity analysis for
'diastolic blood pressure' on 'Ischemic stroke || id:ebi-a-GCST006908'

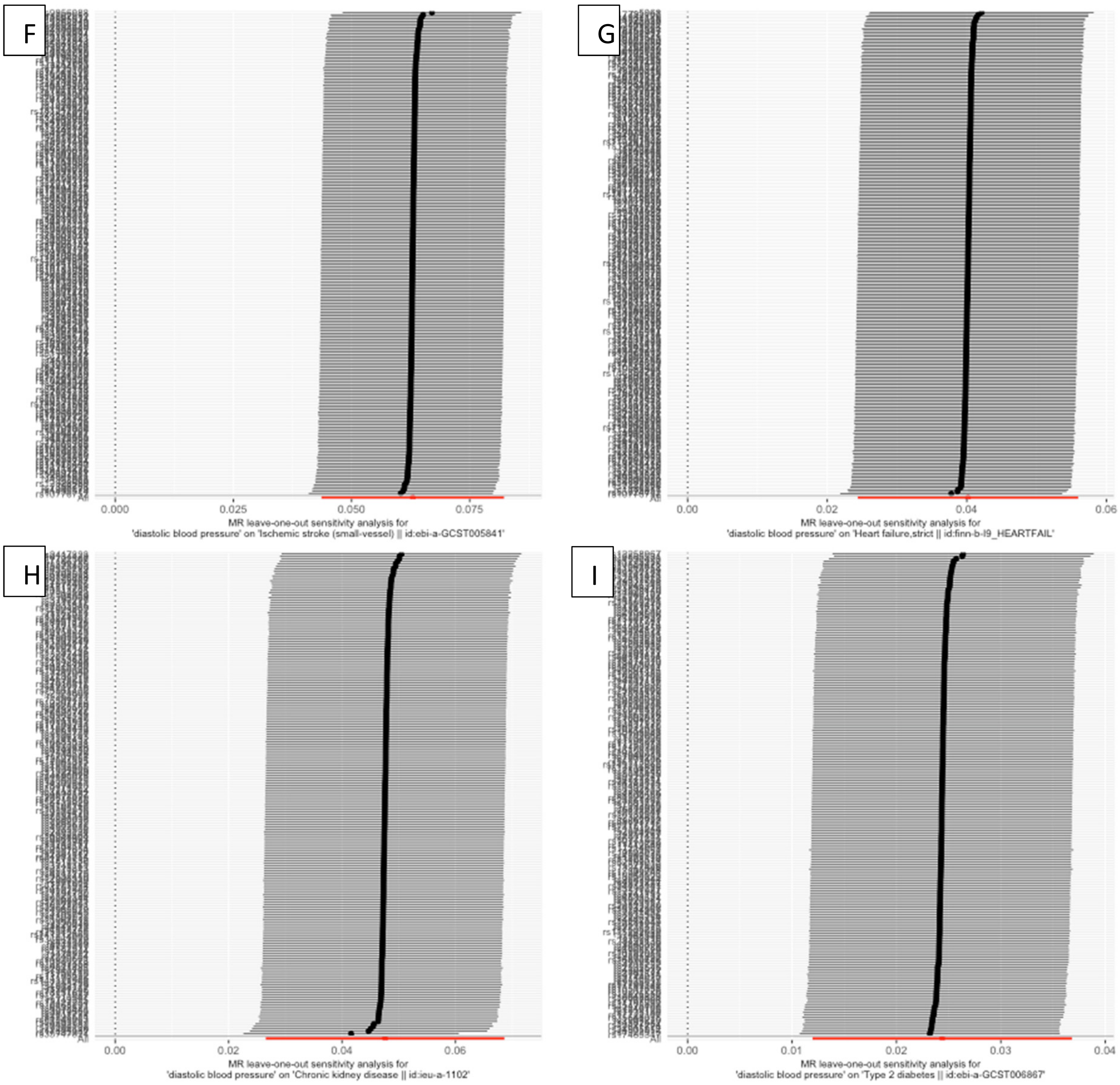

Figure S6. Leave-one-out plots for MR analyses of the causal effect of diastolic blood pressure on (A) atrial fibrillation, (B) coronary artery disease, (C) myocardial infarction, (D) stroke, (E) ischemic stroke, (F) ischemic stroke (small-vessel), (G) heart failure, (H) chronic kidney disease, (I) type 2 diabetes, using inverse-variance weighted, weighted median, and MR Egger methods. SNP = single nucleotide polymorphism, MR = Mendelian randomization.

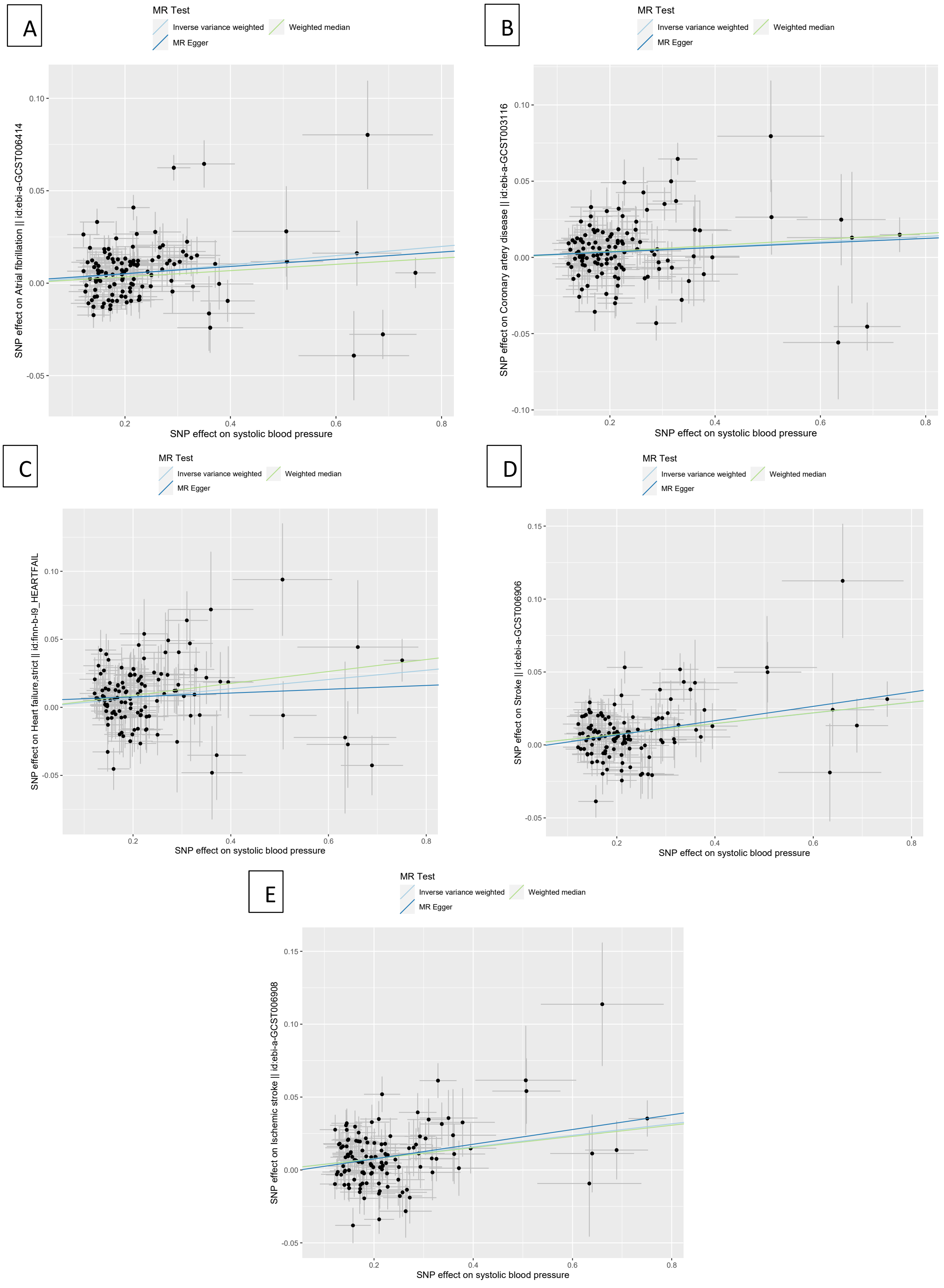
A
MR Test
Inverse variance weighted
MR Egger
Weighted median
SNP effect on Atrial fibrillation || id:ebi-a-GCST006414
SNP effect on systolic blood pressure
B
MR Test
Inverse variance weighted
MR Egger
Weighted median
SNP effect on Coronary artery disease || id:ebi-a-GCST003116
SNP effect on systolic blood pressure
C
MR Test
Inverse variance weighted
MR Egger
Weighted median
SNP effect on Heart failure,strict || id:finn-b-I9_HEARTFAIL
SNP effect on systolic blood pressure
D
MR Test
Inverse variance weighted
MR Egger
Weighted median
SNP effect on Stroke || id:ebi-a-GCST006906
SNP effect on systolic blood pressure
E
MR Test
Inverse variance weighted
MR Egger
Weighted median
SNP effect on Ischemic stroke || id:ebi-a-GCST006908
SNP effect on systolic blood pressure

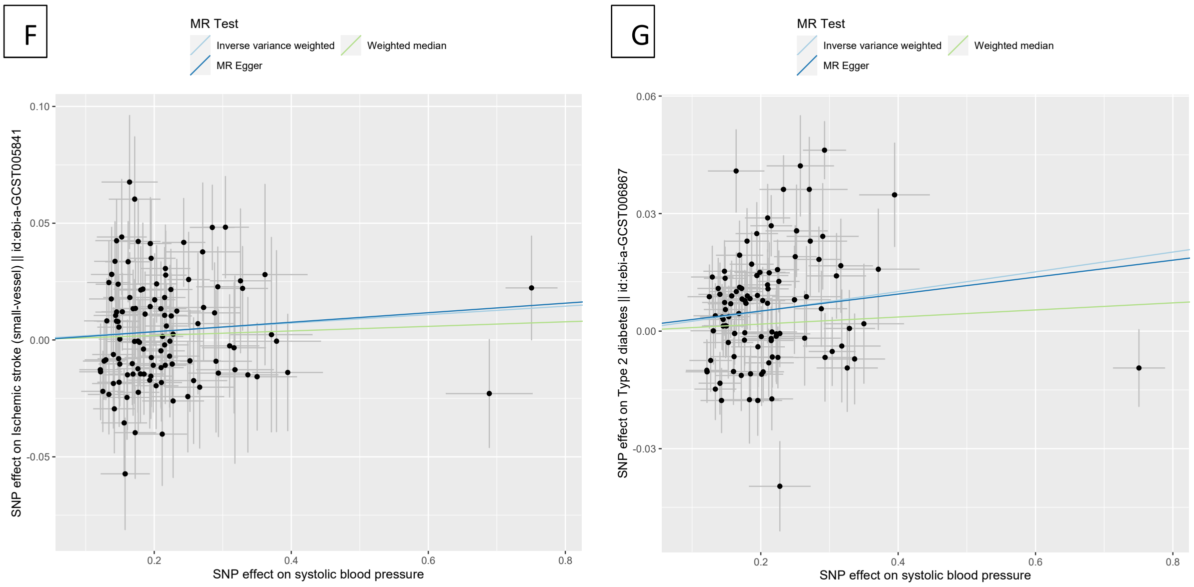

Figure S7. Scatter plots for MR analyses of the independent causal effect of systolic blood pressure on (A) atrial fibrillation, (B) coronary artery disease, (C) heart failure, (D) stroke, (E) ischemic stroke, (F) ischemic stroke (small-vessel), (G) type 2 diabetes, using inverse-variance weighted, weighted median, and MR Egger methods. SNP = single nucleotide polymorphism, MR = Mendelian randomization.

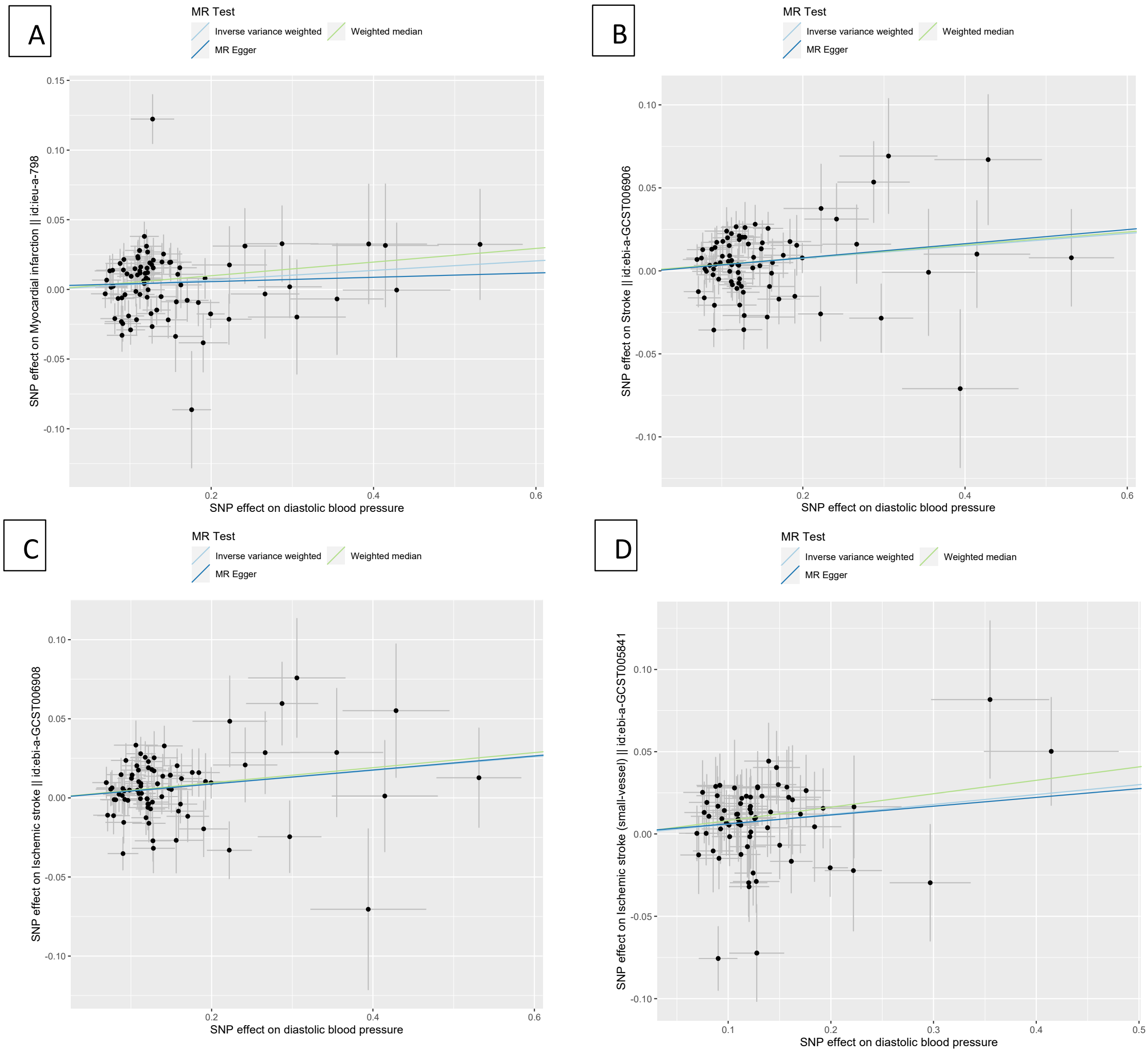

Figure S8. Scatter plots for MR analyses of the independent causal effect of diastolic blood pressure on (A) myocardial infarction, (B) stroke, (C) ischemic stroke, (D) ischemic stroke (small-vessel) using inverse-variance weighted, weighted median, and MR Egger methods. SNP = single nucleotide polymorphism, MR = Mendelian randomization.

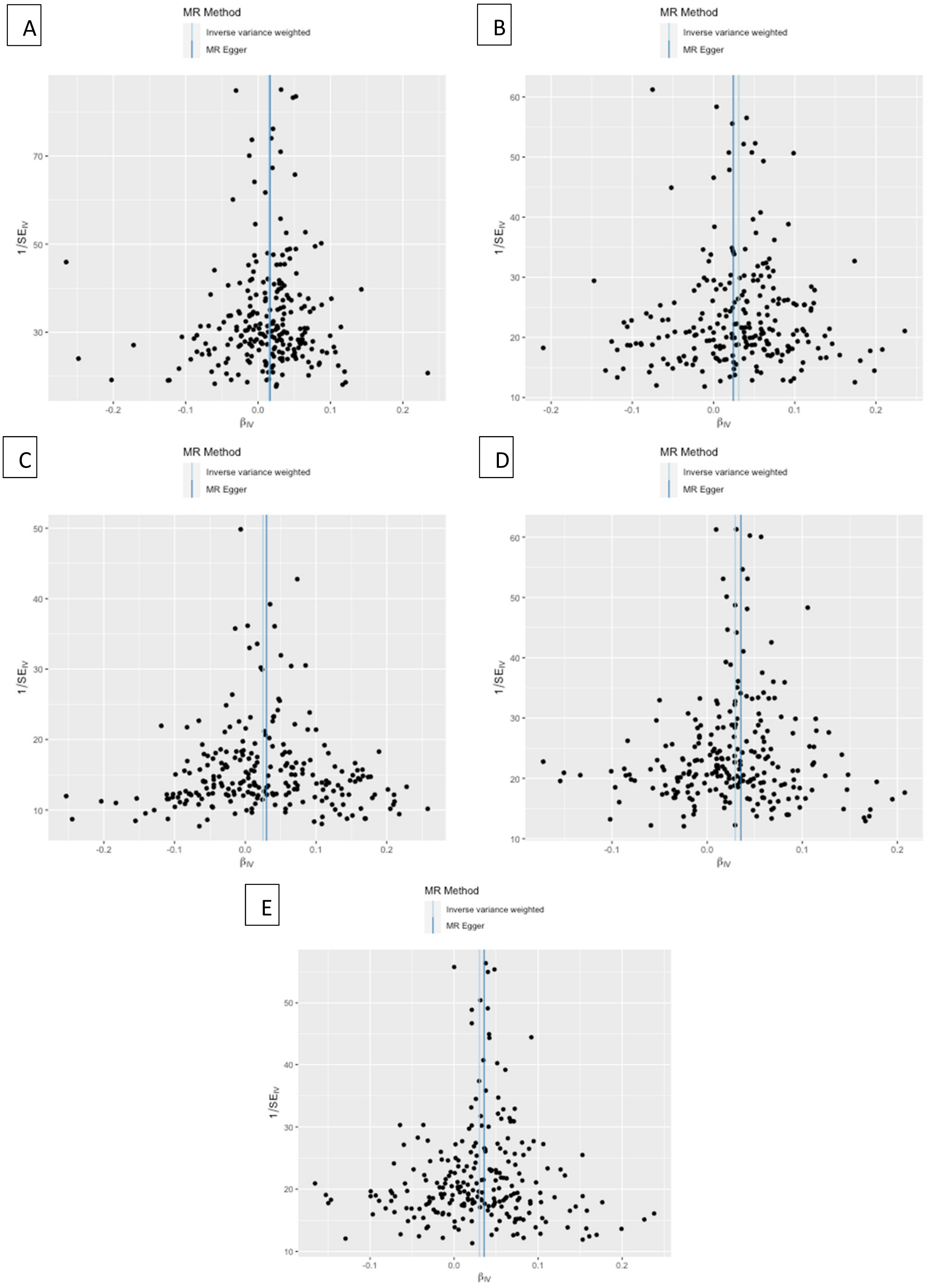
A
B
C
D
E
MR Method
Inverse variance weighted
MR Egger
1/SE_IV
β_IV

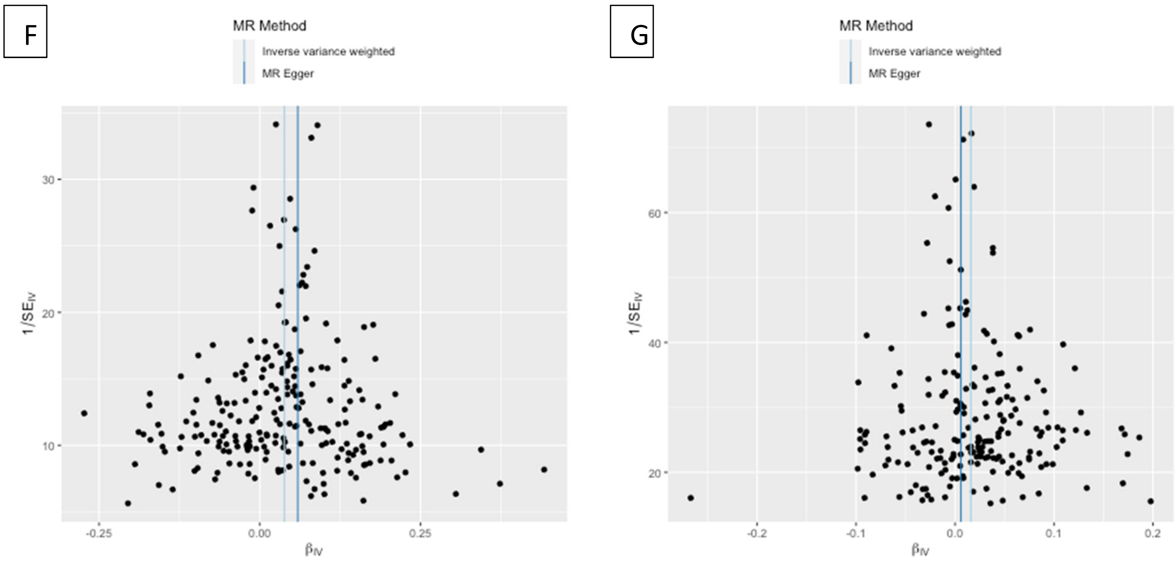

Figure S9. MR-Egger regression funnel plots for systolic blood pressure on (A) atrial fibrillation, (B) coronary artery disease, (C) heart failure, (D) stroke, (E) ischemic stroke, (F) ischemic stroke (small-vessel), (G) type 2 diabetes, using systolic blood pressure-exclusive SNPs. IV = instrumental variable; MR = Mendelian randomization; SE = standard error.

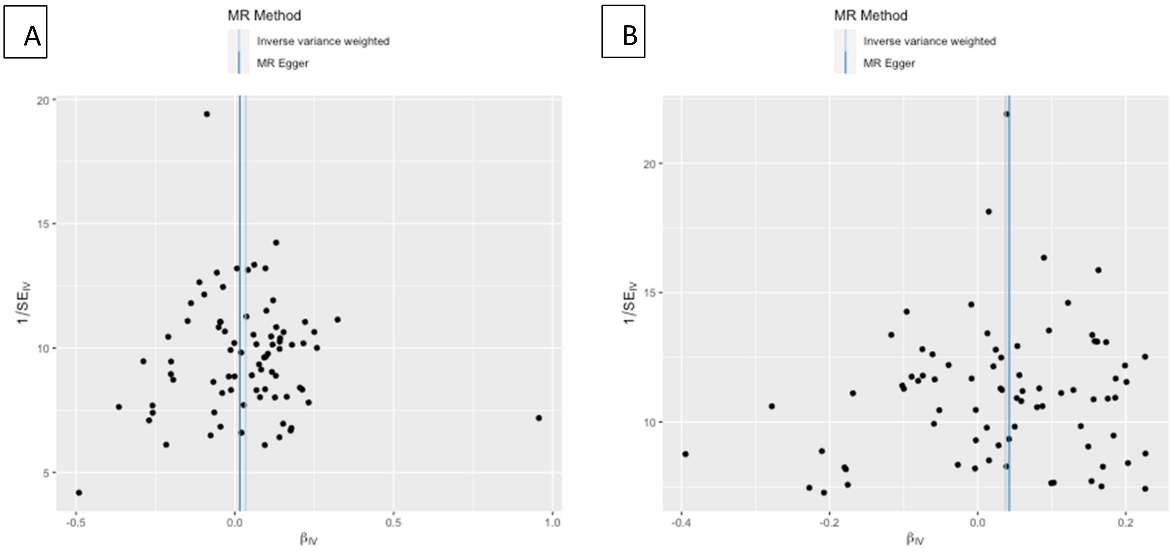

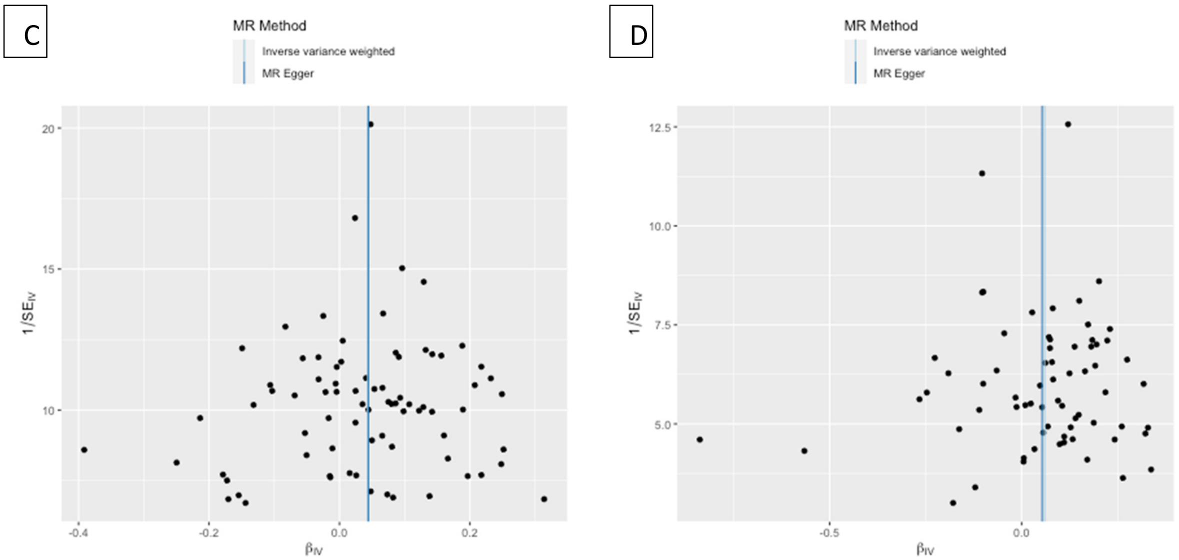

Figure S10. MR-Egger regression funnel plots for diastolic blood pressure on (A) myocardial infarction, (B) stroke, (C) ischemic stroke, (D) ischemic stroke (small-vessel), using diastolic blood pressure-exclusive SNPs. IV = instrumental variable; MR = Mendelian randomization; SE = standard error.

1. Skrivankova VW, Richmond RC, Woolf BAR, Davies NM, Swanson SA, VanderWeele TJ, et al. Strengthening the reporting of observational studies in epidemiology using mendelian randomisation (STROBE-MR): explanation and elaboration. BMJ. 2021;375:n2233.
